# Pre-treatment magnetic resonance imaging in anal cancer: large-scale evaluation of mrT, mrN and novel staging parameters

**DOI:** 10.1038/s41416-024-02759-8

**Published:** 2024-08-21

**Authors:** Hema Sekhar, Rohit Kochhar, Bernadette Carrington, Thomas Kaye, Damian Tolan, Lee Malcomson, Mark P. Saunders, Matthew Sperrin, David Sebag-Montefiore, Marcel van Herk, Andrew G. Renehan

**Affiliations:** 1 Division of Cancer Sciences, School of Medical Sciences, Faculty of Biology, Medicine and Health, University of Manchester, Manchester, UK; 2 Department of Radiology, The Christie NHS Foundation Trust, Manchester, UK; 3 Department of Radiology, St James’ University Hospital, Leeds, UK; 4 Department of Clinical Oncology, The Christie NHS Foundation Trust, Manchester, UK; 5 Division of Informatics, Imaging and Data Sciences, School of Health Sciences, Faculty of Biology, Medicine and Health, University of Manchester, Manchester, UK; 6 Leeds Institute of Medical Research, University of Leeds, St James’s University Hospital, Leeds, UK

**Keywords:** Cancer imaging, Cancer imaging

## Abstract

**Background:**

In patients with squamous cell carcinoma of the anus (SCCA), magnetic resonance (MR) imaging is recommended for pre-treatment staging prior to chemo-radiotherapy (CRT), but large-scale evaluation of its staging performance is lacking.

**Methods:**

We re-characterised pre-treatment MRs from 228 patients with non-metastatic SCCA treated consecutively by CRT (2006–2015) at one UK cancer centre. We derived TN staging from tumour size (mrTr) and nodal involvement (mrN), and additionally characterised novel *beyond TN* features such as extramural vascular invasion (mrEMVI) and tumour signal heterogeneity (mrTSH). Primary outcomes were 5-year overall survival (OS) and 3-year loco-regional failure (LRF). Time-to-event analyses used Kaplan-Meier estimates; Hazard Ratios (HRs) with confidence intervals (CIs) were derived from Cox models.

**Results:**

With a median follow up of 60.9 months, 5-year OS was 74%. Poor OS was associated with increasing mrT (HR: 1.12 per cm [95% CI: 1.07–1.33]), nodal positivity (HR 2.08 [95% CI 1.23–3.52]) and mrEMVI (HR 3.66 [95% CI: 1.88–7.41]). 3-year LRF rate was 16.5%. Increased LRF was associated with increasing mrT (HR: 1.43 per cm [95% CI: 1.26–1.63]), nodal positivity (HR 2.70 [95% CI 1.39–5.24]) and mrTSH (HR 2.66 [95% CI 1.29–5.48]).

**Conclusions:**

In SCCA, the study demonstrates that mrT and mrN stages are prognostic, while mrEMVI and mrTSH may be novel prognostic factors.

## Introduction

In patients with squamous cell carcinoma of the anus (SCCA), chemoradiotherapy (CRT) is the mainstream of treatment [1] but is associated with high rates of treatment-related morbidity [2] with 15–20% subsequently developing local disease failure [3]. As pre-treatment T- and N-stage are key determinants of local disease failure and survival [4], accurate measurement of these stages is important for treatment planning and prognostication. Magnetic resonance (MR) is the key imaging modality for pre-treatment staging as recommended in the UK [5], European [6], and Australian [7] clinical guidelines, though notably, US guidelines offer a range of pre-treatment staging modalities including MR, CT, and endoanal ultrasound. However, the studies evaluating pre-treatment staging and prognosis, to date, have been from single-institute series [8–11], national cancer databases [12] and secondary analyses of trial data [13, 14], in which staging was based on combinations of clinical examination, CT-imaging, endoanal ultrasound and/or chest-X-rays, but not MR imaging. Studies have evaluated the role of pre-treatment MR imaging in SCCA, but these have been small-scale studies (typically less than 100 patients) [15–19], mixed histological types [20] and demonstrated potential clinical utility rather than at scale prognostic evaluation. Furthermore, the evidence underpinning the currently used American Joint Committee on Cancer (AJCC) staging systems for SCCA (the 8th edition from 2017, and now, the very recent 2023 9th edition) is derived from the RTOG 98-11 trial [13, 14], where MR imaging was not mandated.

Until recently, CRT has been prescribed as a ‘one-size’ fits all approach, but dose reduction or escalations of radiotherapy (RT), based on differential local failure risks—a *personalised treatment approach*—might result in a better toxicity profile without compromising oncological outcome [21]. The current UK-based PLATO (PersonaLising Anal cancer radiotherapy dOse) [22] trial addresses this hypothesis and is evaluating the strategy of stratified RT dosing based on very low risk (anal margin, T1N0); low risk (mrT <4 cm, N0); and high-risk disease (T2–T3, N1–3; T4, any *N*), and with a target recruitment of 929 patients, is presently the largest anal cancer trial worldwide. Pre-treatment MR imaging is mandated in PLATO. Other currently recruiting anal cancer trials mandating MR imaging include the RADIANCE trial [23] and a Chinese CRT with and without PD-1 blockade trial [24]. For these and future personalised treatment approach trials to translate into clinical practice, there is a need to evaluate at scale the prognostic role of MR staging in patients with SCCA.

Within a large institute-based treatment series, the aims of this study were fourfold. First, to validate the current AJCC TNM (tumour and nodal) system using pretreatment mrTNM against oncological outcomes, namely survival and local disease failure. Second, to explore the prognostic utility of *beyond TNM* MR characteristics, for example, extramural vascular invasion and tumour signal heterogeneity. Third, to test the reproducibility of the above MR-based parameters across radiologists, for generalisability of results. Fourth, noting that the nodal sub-classification in AJCC TNM 8th edition (2017) [25] differed substantially to that used in the AJCC TNM 7th edition (2009) [26], to test the performance characteristics of the TNM staging systems derived from 8th versus 7th editions.

## Methods

### Patients

From a prospectively collected database [3], we identified consecutive patients with non-metastatic SCCA treated by CRT with curative intent at the Christie NHS Foundation Trust, Manchester, United Kingdom, who had MR imaging as routine pre-treatment MR staging, between 1st November 2006 and 31st January 2015, with follow-up until 30th April 2022. The CRT treatment protocol has been described elsewhere [3] and pre-dated the routine use of IMRT.

### MR imaging

All MR imaging was performed on ≥1.5 Tesla MR employing pelvic phase-array body coils (acquisition protocol in Table S1). For inclusion in this analysis, scans comprised multiplanar small field of view high-resolution T2-weighted sequences and a large axial field of view series of the pelvis (either T1 or T2-weighted). Patients with squamous cell carcinoma were excluded if the tumour epicentre was in the rectum or if the anatomy was significantly distorted due to prior surgery. Images were re-characterised by two consultant radiologists (RK and BC, 15 and 25 years’ experience, respectively), blinded to patient outcomes. From 2011, pre-treatment staging also included Fluoro-Deoxy-Glucose Positron Emission Tomography/Computed Tomography—for this study, the radiologists were blinded to reports from these scans.

### Tumour characterisations

The detailed definitions of the MR-derived tumour characteristics are described in Supplementary material. Tumours located above the anal verge were classified as canal tumours; those entirely below the verge as margin tumours (Fig. S1) [27]. The anterior-posterior (AP), left-right (LR) and cranio-caudal (CC) size were recorded to determine the longest diameter, which was taken as the mrT (Fig. S2) [25]. We explored whether other stratification of tumour size might be prognostic, and specifically stratified into a binary category of mrT <4 cm and ≥4 cm as used in the current large UK PLATO trial and hypothesised to be prognostic for loco-regional failure [22].

We classified lymph node involvement if either or both of the following two morphological criteria were met: (i) signal heterogeneity within the node, representative for necrosis; and (ii) border irregularity, representative of extracapsular tumour invasion (Fig. S3) [28]. Nodal fields were separated into two midline fields: (i) mesorectal and (ii) presacral; and three bilateral fields: (iii) internal iliac; (iv) inguinal; and (v) external iliac (Table S2) [7, 29]. From the above T- and N- features, we derived the stage according to AJCC 7th and 8th editions (Table S3). (During the undertaking of this study, the AJCC 9th edition was published in early 2023 [30], which recognised that overall survival using AJCC 8th edition stage groupings lacked a hierarchical order in which stage IIIA (T1-T2N1M0) anal cancer was associated with a better prognosis than stage IIB (T3N0M0) disease. This was addressed in the updated edition by redefining the stage groupings but, in terms of T and N classifications, these are the same for AJCC 8th and 9th editions).

Other tumour characteristics identified were: (i) MR-defined extramural vascular invasion (mrEMVI, Fig. S4) [31]; (ii) MR-defined tumour signal heterogeneity (mrTSH, Fig. S5); (iii) sphincter infiltration (Fig. S6); (iv) sepsis (Fig. S7); and (v) organ extension [25].

### Reproducibility and generalisability

Reproducibility was assessed by estimating intra- and inter-observer variability for mrT, N positivity, number of positive nodes, mrEMVI and mrTSH, in 20 patients treated at the Christie. Generalisability was assessed between two radiologists at the Christie Foundation Trust (RK and BC) and one radiologist from St James’s University Hospital, Leeds, United Kingdom (TK) in 18 patients treated at the Christie. Agreement was expressed using Kappa statistics—level of agreement descriptive terms were by Kappa scores 0–0.20, no; 0.21–0.39, minimal; 0.40–0.59, weak; 0.60–0.79, moderate; 0.80–0.90; strong; and >0.90, almost perfect [32]. Inter-concordance correlation (ICC) scores were applied for continuous variables and similar descriptive terms for agreement used [33].

### Statistical analysis

Stata software, version 15 (Stat Corp., TX, USA) was used for all statistical analyses. The primary outcomes were (i) 5-year overall survival (OS), defined as the period of time until death from any cause from the date of treatment initiation, and (ii) 3-year loco-regional failure (LRF), defined as residual or recurrent disease in the pelvis within the initial RT field. Criteria for establishing LRF included histological confirmation, clinically palpable disease and radiographically evident disease. The secondary outcome was 5-year distant metastatic failure (DMF), defined as failure at any disease sites considered to be distant, including common iliac nodes and distant organ involvement such as liver, lung or bone. We derived Kaplan-Meier (K-M) estimates, with 95% confidence intervals (CI), and we assessed for differences across pre-treatment MR characteristics by performing univariable and multivariable analyses using Cox models. *P* values less than 0.05 were considered to be statistically significant. Codes are available on request.

We sought to compare the clinical usefulness of the 7th and 8th AJCC TNM staging systems. We initially described summary statistics, for example, proportions per staging group and then evaluated performance characteristics by deriving the C-statistics, and their 95% confidence intervals, from Cox models [34]. We defined clinical usefulness as a mean difference greater than 0.05 and statistically significant.

## Results

We identified pre-treatment staging MR scans in 315 patients. After the exclusion of scans that did not meet the minimum quality criteria; palliative, metastatic and radiotherapy-only treated cases, and Tx stage tumours, there were 228 analysable scans (Fig. S8).

### Patients and tumour characteristics

The baseline characteristics are detailed in Table 1. mrT2 was the predominant stage with a corresponding median mrT of 4.2 cm (IQR: 3.2 cm to 5.6 cm). Forty-five patients (19.7%) presented with invasion to adjacent organs, i.e. T4 disease, including the vagina (*N*: 37), prostate (*N*: 7), and prostate and penis (*N*: 1). Most patients (91.2%) had anal canal disease or canal disease extending into the margin, tumour straddling the dentate line (61.4%) and over half of the cohort had tumours that extended into the rectum (61.4%). mrTSH was seen in 51.3% of patients, while only 5.7% and 8.3% of patients demonstrated mrEMVI or sepsis respectively. Extension into rectum, sphincter infiltration, mrTSH and mrEMVI were associated with increasing tumour size (*p* < 0.0001, *p* < 0.0001, *p* = 0.030 and *p* = 0.0004, respectively) (Table S3). Nodes were detected in 92 (40.3%) patients. There were five patients with external iliac nodes; this group was not included in the time-to-event analyses.

**Table 1:** Baseline patient and tumour characteristics of 228 patients with analysable pre-treatment MR scans, Christie series 2006 to 2015, stratified by T size < and ≥ 4 cm

	Totals	Stratification by PLATO trial T size strata		*P* value
		<4 cm (102)	≥4 cm (126)	
Demographics				
Men (%)	86 (37.7)	41 (40.2)	45 (35.7)	0.49^a^
Age (years, median, IQR)	61 (52–69)	60 (51–68)	62 (52–70)	0.45^c^
MSM (%)	18 (7.9)	3 (2.9)	15 (11.9)	0.007^a^
HIV positive (%)	9 (4.0)	2 (2.0)	7 (5.6)	0.19^b^
Tumour Characteristics				
Anatomical Position (%)				
Canal	173 (75.9)	73 (71.6)	100 (79.4)	
Margin	20 (8.8)	16 (15.7)	4 (3.2)	
Canal and Margin	35 (15.4)	13 (12.8)	22 (17.5)	0.003^a^
T-Size (cm, median, IQR)	4.2 (3.2–5.6)	3.0 (2.5–3.5)	5.5 (4.6–6.3)	<0.0001^c^
T-Stage (%)				
T1	7 (3.1)	7 (6.7)	0	
T2	124 (54.4)	88 (86.3)	36 (28.6)	
T3	52 (22.8)	0	52 (41.3)	
T4	45 (19.7)	7 (6.7)	38 (30.2)	<0.001^b^
Nodal Characteristics				
Any nodal detection (%)	92 (40.2)	26 (25.5)	66 (52.4)	<0.001^a^
Perirectal Nodes (%)	62 (27.2)	14 (27.7)	48 (38.1)	<0.001^a^
Pre-Sacral Nodes (%)	24 (10.5)	5 (4.9)	19 (15.1)	0.013^a^
Inguinal Nodes (%)	33 (14.5)	9 (8.8)	24 (19.1)	0.029^a^
Internal Iliac Nodes (%)	18 (7.9)	3 (2.9)	15 (11.9)	0.013^a^
External Iliac Nodes (%)	5 (2.2)	1 (1.0)	4 (3.2)	0.38^b^
Total Nodes (median, IQR)	0 (0–1)	0 (0–1)	1 (0–3)	<0.0001^c^
N Stage (AJCC V7)				
N0 (%)	136 (59.7)	76 (74.5)	60 (47.6)	
N1 (%)	44 (19.3)	13 (12.8)	31 (24.6)	
N2 (%)	20 (8.8)	8 (7.8)	12 (9.5)	
N3 (%)	28 (12.3)	5 (4.9)	23 (18.3)	<0.001^a^
Additional Characteristics				
Dentate Position (%)				
Above	36 (15.8)	22 (21.6)	14 (11.1)	
Below	52 (22.8)	38 (37.3)	14 (11.1)	
Straddling	140 (61.4)	42 (41.2)	98 (77.8)	<0.001^a^
Rectal Extension (%)	140 (61.4)	43 (42.2)	97 (77.0)	<0.001^a^
mrEMVI (%)	13 (5.7)	2 (2.0)	11 (8.7)	0.028^a^
mrTSH (%)	117 (51.3)	49 (48.0)	68 (54.0)	0.37^a^
Sphincter Infiltration^d^ (%)				
Confined to Internal Sphincter	62 (27.4)	44 (44.0)	18 (14.3)	
Confined to External Sphincter	113 (50.0)	47 (47.0)	66 (52.4)	
Breached into ischio-rectal fossa	51 (22.6)	9 (9.0)	42 (33.3)	<0.001^a^
Perianl abscess (%)	19 (8.3)	9 (8.8)	10 (7.9)	0.81^a^
Organ Extension (%)	45 (19.7)	7 (6.7)	38 (30.2)	<0.001^a^

*N* number of patients, *IQR* interquartile range, *MSM* men who have sex with men, *EMVI* extramural vascular invasion, *TSH* tumour signal heterogeneity

^a^Chi-squared test;

^b^Fisher’s exact test;

^c^Wilcoxan Rank Sum test;

^d^Readers were unable to assign the degree of sphincter infiltration in two cases and of the 226 further assessable patients, half had tumours that had breached beyond the internal sphincter but remained confined within the external sphincter and the other quarter had tumours that had breached the external sphincter and infiltrated into the ischio-anal fossa.

### Reproducibility and generalisability

Intra- and inter-observer agreement results are presented in Table 2. For mrT, intra-observer agreement was strong (ICC: 0.86), whilst within-institutional and between-institutional inter-observer agreements ranged from moderate to strong (ICC: 0.73 to 0.88). For nodal involvement, intra-observer agreement was perfect (kappa: 1), whilst within-institutional inter-observer agreement was strong (kappa: 0.89), and between institutional inter-observer agreement was weak to moderate (kappas: 0.53 to 0.66). Intra- and inter-observer agreements for the total number of nodes detected ranged from moderate to strong (ICCs: 0.50 to 0.89).

**Table 2:** Same- and between-institute intra- and inter-radiologist variation of tumour and nodal parameters

	Same institute (*n*: 20)		Between institute (*n*: 18)	
	Intra-radiologist (95% CI)	Inter-radiologist model A (95% CI)	Inter-radiologist model B (95% CI)	Inter-radiologist model C (95% CI)
Tumour Parameters				
mrT-Size^a^	0.86 (0.73–0.98)	0.73 (0.50–0.95)	0.88 (0.77–0.98)	0.82 (0.66–0.98)
mrEMVI^b^	0.64 (0.006–1.0)	n/a	n/a	−0.08 (-0.24–0.08)
mrTSH^b^	0.89 (0.67–1.0)	0.57 (0.24–0.91)	0.55 (0.16–0.94)	0.14 (-0.27–0.55)
Sphincter Infiltration^b^	0.40 (0.03–0.64)	0.25 (0.10–0.40)	0.50 (0.02–0.81)	−0.024 (-0.43–0.38)
Nodal Parameters				
Nodal Involvement^b^	1.00 (1–1)	0.89 (0.67–1.0)	0.66 (0.31–1.0)	0.53 (0.13–0.93)
Total Number of Nodes^a^	0.95 (0.90–0.99)	0.95 (0.91–1.0)	0.89 (0.78–0.99)	0.78 (0.60–0.97)
N Stage^b^	0.89 (0.53–1.0)	0.72 (0.37–1.0)	0.63 (0.41–0.91)	0.50 (0.15–0.78)

Inter-radiologist model A is between the two radiologists (RK and BC) from the Christie Foundation Trust; Inter-radiologist model B is between radiologist 1 from The Christie and the radiologist from Leeds University Teaching Hospitals, LTHT (RK and TK) and Inter-radiologist model C is between second radiologist from The Christie and the radiologist from LTHT (BC and TK).

*CI* confidence interval, *EMVI* extramural venous invasion, *TSH* tumour signal heterogeneity.

^a^Inter-concordance Correlation (ICC) score;

^b^Kappa statistic.

Intra-observer agreement for mrEMVI was moderate (kappa: 0.64), whilst that for mrTSH was strong (kappa: 0.89). However, there was no inter-observer agreement for these two characteristics (kappa: −0.08 to 0.14). There was also no inter- and intra-observer agreement for sphincter involvement.

### Overall survival

**Fig. 1:**
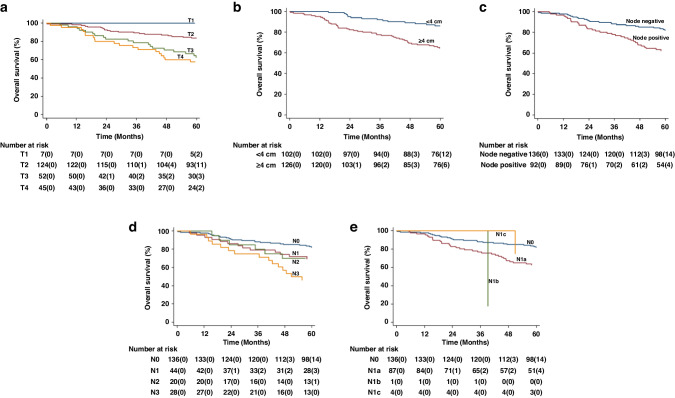
Overall Survival. K-M plots by: **a** mrT stage, **b** mrT(4 cm), **c** mrN, **d** mrN Stage AJCC 7th edition and **e** mrN Stage AJCC 8th edition. The values in parentheses are numbers censored.

With a median follow-up time of 60.9 months (IQR: 50.0–60.9 months), there were 58 deaths (25.4%) with a 5-year OS rate of 74.2% (95% CI: 67.9–79.4%). The 5-year OS rates reduced in a stepwise manner from T1 to T4 tumours (Table 3, Fig. 1), although statistically significant differences were only seen between tumours of stages T1 and T2 and tumours of stages T3 and T4 in Cox models both unadjusted and adjusted for nodal involvement. mrT, as a continuous variable adjusted for nodal status, was associated with OS with an HR of 1.21 (95% CI: 1.07 to 1.36) per cm. The 5-year OS rates were reduced significantly where mrT ≥ 4 cm (<4 cm vs ≥4 cm: 86.1% [95% CI: 77.6% to 91.5%] vs 64.6% [55.5% to 72.3%]; HR 2.52 [95% CI: 1.36 to 4.67] *p* = 0.003).

**Table 3:** Association of MR-derived tumour characteristics with overall survival and locoregional failure and LRF in 228 patients with analysable pre-treatment MR scans, Christie series 2006 to 2015

Variable	Overall survival					Loco-regional failure				
	5 yr OS (95% CI)	HR^a^ (95% CI)	*p* value	HR^b^ (95% CI)	*p* value	3 yr LRF (95% CI)	HR^a^ (95% CI)	*p* value	HR^b^ (95% CI)	*p* value
Position										
Canal (208)	72.3 (65.5–77.9)	Referent		Referent		18.2 (13.-5–24.2)	Referent		Referent	
Margin (20)	94.7 (68.1–99.2)	0.16 (0.02–1.16)	0.070	0.23 (0.03–1.69)	0.15	0	0.24 (0.03–1.71)	0.153	0.37 (0.05–2.70)	0.326
T Stage										
T1 (7)	100	0^d^	1.00	0^d^	1.00	0^d^	0^d^	1.00	0^d^	.
T2 (124)	83.7 (75.9–89.2)	Referent		Referent		6.5 (3.3–12.6)	Referent		Referent	
T3 (52)	62.5 (47.6–74.2)	2.57 (1.37–4.82)	0.0030	2.28 (1.20–4.31)	0.011	29.0 (18.6–43.5)	4.42 (2.00–9.74)	<0.001	3.72 (1.67–8.30)	<0.001
T4 (45)	57.7 (42.0–70.6)	3.10 (1.66–5.82)	<0.001	2.60 (1.36–4.97)	0.0040	32.9 (18.6–43.5)	4.49 (1.99–10.11)	<0.001	3.38 (1.46–7.81)	0.004
Size (per cm) (228)		1.23 (1.11–1.38)	<0.001	1.21 (1.07–1.36)	0.0010		1.45 (1.29–1.64)	<0.001	1.43 (1.26–1.63)	<0.001
T size with cutoff at 4 cm										
<4 (102)	86.1 (77.6–91.5)	Referent		Referent		4.9 (2.1–11.4)	Referent		Referent	
≥4 (126)	64.6 (55.5–72.3)	2.97 (1.63–5.42)	<0.001	2.52 (1.36–4.67)	0.003	26.3 (19.4–35.1)	6.56 (2.57–16.75)	<0.001	5.17 (1.99–13.44)	<0.001
Any Nodes										
Negative (136)	82.1 (74.5–87.6)	Referent		Referent		9.0 (5.2–13.4)	Referent		Referent	
Positive (92)	62.5 (51.6–71.5)	2.36 (1.40–3.98)	<0.001	2.08 (1.23–3.52)	0.006	27.6 (19.5–38.0)	3.41 (1.76–6.61)	<0.001	2.70 (1.39–5.24)	0.003
Total Nodes		1.19 (1.10–1.28)	<0.001	1.16 (1.07–1.26)	<0.001		1.18 (1.07–1.30)	<0.001	1.13 (1.02–1.26)	0.016
N stage V7										
N0 (136, 60%)	82.1 (74.5–87.6)	Referent		Referent		9.0 (5.2–15.4)	Referent		Referent	
N1 (44, 19%)	69.6 (53.4–81.1)	1.85 (0.94–3.64)	0.074	1.72 (0.87–3.37)	0.117	27.4 (16.6–43.2)	3.38 (1.56–7.29)	0.002	2.91 (1.35–6.29)	0.007
N2 (20, 9%)	70.0 (45.1–85.3)	1.81 (0.74–4.44)	0.192	1.44 (0.58–3.58)	0.43	25.3 (11.4–50.6)	2.78 (0.99–7.80)	0.052	1.78 (0.62–5.12)	0.285
N3 (28, 12%)	46.4 (27.6–63.3)	3.67 (1.92–7.00)	<0.001	3.24 (1.70–6.20)	<0.001	29.4 (15.9–50.3)	3.97 (1.70–9.30)	<0.001	3.22 (1.38–7.54)	0.007
N stage V8										
N0 (136, 60%)	82.1 (74.5–87.6)	Referent		Referent		9.0 (5.2–15.4)	Referent		Referent	
N1a (87, 38%)	69.6 (53.4–81.1)	1.85 (0.94–3.64)	0.074	1.72 (0.87–3.37)	0.117	29.2 (20.7–40.1)	3.64 (1.88–7.07)	<0.001	2.96 (1.52–5.75)	<0.001
N1b (1. 0.4%)	70.0 (45.1–85.3)	1.81 (0.74–4.44)	0.192	1.44 (0.58–3.58)	0.434	-	-	-	-	-
N1c (4, 2%)	46.4 (27.6–63.3)	3.67 (1.92–7.00)	<0.001	3.24 (1.70–6.20)	<0.001	-	-	-	-	-
Dentate–Position										
Above (36)	86.0 (69.6–93.9)	Referent		Referent		11.1 (4.3–27.0)	Referent		Referent	
Straddling (140)	67.5 (59.0–74.6)	2.50 (1.00–6.31)	0.052	2.09 (0.81–5.38)	0.128	19.9 (14.1–27.7)	1.51 (58.1–3.90)	0.400	0.89 (0.33–2.39)	0.823
Below (52)	84.4 (71.2–91.9)	1.11 (0.36–3.39)	0.856	1.36 (0.44–4.23)	0.54	11.6 (5.4–24.0)	0.97 (0.31–0.05)	0.956	1.11 (0.34–3.59)	0.868
Rectal Extension										
No (88)	82.6 (72.7–89.1)	Referent		Referent		10.4 (5.5–19.0)	Referent		Referent	
Yes (140)	69.0 (60.5–76.0)	1.99 (1.10–3.58)	0.022	1.51 (0.83–2.75)	0.173	20.5 (14.6–28.3)	1.76 (0.88–3.52)	0.111	1.20 (0.60–2.43)	0.607
Extra-mural Vascular Invasion										
No (215)	72.4 (71.1–82.4)	Referent		Referent		14.6 (10.5–20.2)	Referent		Referent	
Yes (13)	20.5 (3.9–46.3)	5.24 (2.64–10.41)	<0.001	3.66 (1.80–7.41)	<0.001	51.4 (27.0–80.8)	4.11 (1.72–9.82)	<0.001	2.31 (0.95–5.61)	0.064
Tumour Signal Heterogeneity										
No (111)	80.7 (72.0–87.0)	Referent		Referent		7.4 (3.8–14.2)	Referent		Referent	
Yes (117)	68.0 (58.6–75.7)	1.89 (1.11–3.23)	0.020	1.69 (1.00–2.90)	0.057	25.3 (18.3–34.3)	3.22 (1.57–6.58)	<0.001	2.66 (1.29–5.48)	0.008
Sphincter Infiltration^c^										
Internal (62)	78.7 (66.2–87.0)	Referent		Referent		8.2 (3.5–18.6)	Referent		Referent	
External (113)	72.9 (63.5–80.2)	1.27 (0.66–2.43)	0.47	0.82 (0.41–1.63)	0.57	13.5 (8.4–21.4)	1.51 (0.59–3.85)	0.391	0.85 (0.33–2.23)	0.747
Ischio-anal fossa(51)	70.5 (55.9–81.0)	1.46 (0.69–3.06)	0.32	0.72 (0.31–1.65)	0.434	34.0 (22.7–48.9)	4.04 (1.60–10.18)	0.003	1.46 0.54–3.97)	0.456
Perianal abscess										
No (209)	73.3 (66.7–78.9)	Referent		Referent		17.5 (13.0–23.5)	Referent		Referent	
Yes (19)	83.9 (57.9–94.5)	0.59 (0.18–1.88)	0.370	0.58 (0.18–1.84)	0.353	5.6 (0.8–33.4)	0.27 (0.04–2.00)	0.201	0.25 (0.03–1.84)	0.175
Primary Organ Extension										
No (220)	78.3 (71.5–83.7)	Referent		Referent		12.7 (8.6–18.5)	Referent		Referent	
Yes (45)	57.7 (42.0–70.6)	2.27 (1.31–3.94)	0.003	1.57 (0.88–2.78)	0.124	32.9 (20.9–49.1)	2.45 (1.28–4.70)	0.007	1.37 (0.71–2.65)	0.346

Values in parentheses in the ‘Variable’ column represent the number of patients; T2 has been selected as the referent for T-stage analyses as it represents the most frequent stage at presentation

*OS* overall survival, *HR* hazard ratio, *CI* confidence interval;

^a^Univariable Cox Regression;

^b^Multivariable Cox regression adjusted for T-size and nodal status (T-stage and T-size only adjusted for nodal status);

^c^Parameter not assessable in two patients, therefore analysis in 226 patients;

^d^No events occurred.

There were no associations with OS for rectal extension, infiltration into the ischio-anal fossa, tumour position relative to the dentate line, and presence of sepsis.

The 5-year OS rates were reduced significantly in the presence of nodal involvement (negative vs positive: 82.1% [95% CI: 74.5% vs 87.6%] vs 62.5% [95% CI: 51.6% to 71.5%]). Although 5-year OS rates reduced with increasing AJCC 7th edition N-stage (N0: 82.1% [95% CI: 74.5% to 87.6%]; N1: 69.6% [95% CI: 53.4% to 81.1%]; N2: 70.0% [95% CI: 45.1% to 85.3%; N3: 46.4% [95% CI: 27.6% to 63.3%]), adjusting for mrT, left only the N3 group as independently prognostic (Table 3). The total number of nodes detected was associated with OS after adjustment for mrT (HR 1.16 [95% CI: 1.07 to 1.26]). Applying AJCC 8th edition N-stage reflected the above with only N1c remaining significantly associated (Table 3). There was a trend for reducing 5-year OS by nodal location as follows perirectal without pre-sacral nodes, 70.1%; inguinal nodes, 57.6%; pre-sacral nodes (with or without perirectal nodes), 50.0%; and internal iliac nodes, 44.4% (Table S4).

**Fig. 2:**
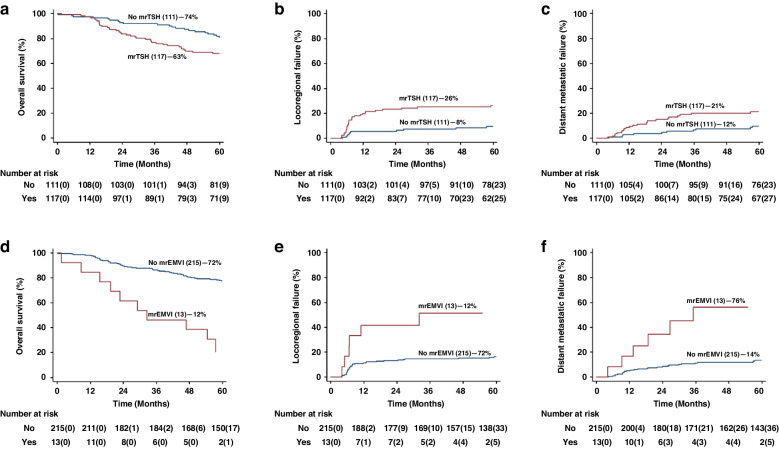
Overall survival (OS), loco-regional failure (LRF) and distant metastatic failure (DMF) for tumour signal heterogeneity (mrTSH) and extramural vascular invasion (mrEMVI). K-M plots by **a** OS for mrTSH; **b** LRF for mrTSH; and **c** DMF for mrTSH; **d** OS for mrEMVI; **e** LRF for mrEMVI; and **f** DMF for mrEMVI. The values in parentheses are numbers censored.

mrEMVI was present in 5.7% of patients being exclusively in tumours exhibiting rectal extension, where it  represented 9.3% of tumours. The presence of mrEMVI was strongly associated with lower 5-year OS (mrEMVI negative vs positive: 72.4% [95% CI: 71.1% to 82.4%] vs 20.5% [95% CI: 3.9% to 46.3%]) (Fig. 2), with its association persisting when adjusting for mrT and nodal status (HR 3.66 [95% CI 1.80 to 7.41]). mrTSH was associated with a 12% lower rate of 5-yr OS, but this association lost statistical significance when adjusting for mrT and nodal status.

### Loco-regional failure

**Fig. 3:**
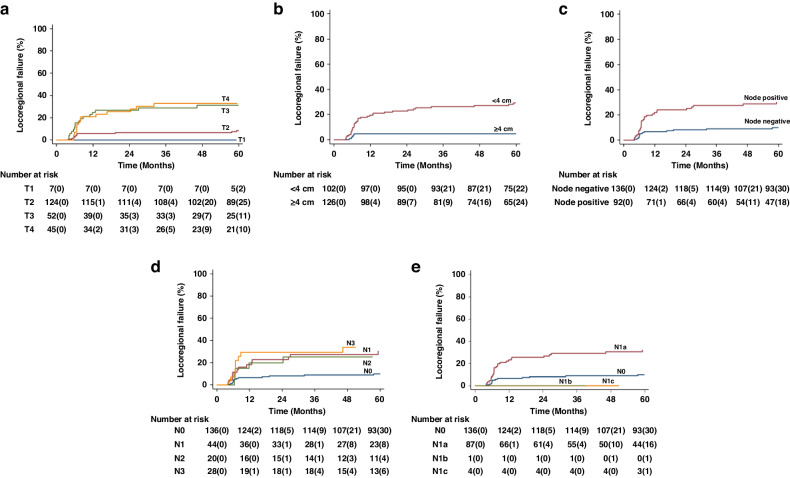
Loco-regional failure. K-M plots by **a** mrT stage, **b** mrT(4cm), **c** mrN, **d** mrN Stage AJCC 7th edition and **e** mrN Stage AJCC 8th edition. The values in parentheses are numbers censored.

There were 40 (17.5%) LRF events with an actuarial 3-year LRF rate of 16.6% (95% CI: 12.3% to 22.1%). The 3-year LRF rates increased in a stepwise manner from T1 to T4 tumours (Table 3, Fig. 3). Despite an increase in 3-year LRF with T-stage, those with T1 and T2 demonstrated similar outcomes to each other, compared with the higher rates of 3-year LRF demonstrated by the T3 and T4 tumours in Cox regression. Tumour size, as a continuous variable, was associated with OS with an HR of 1.43 per cm (95% CI 1.26 to 1.63), *p* < 0.001. The 3-year LRF rates were increased significantly where mrT ≥ 4 cm (26.3% [95% CI: 19.4% to 35.1%] vs 4.9% [95% CI: 2.1% to 11.4%], *p* < 0.001).

None of the tumour position parameters were associated with LRF. Extension to the ischio-anal fossa was associated with high 3-year LRF rates, as was organ extension, but these associations did not persist after adjusting for mrT and nodal status.

The 3-year LRF rates were increased in the presence of nodal involvement (negative vs positive: 9.0% [95% CI: 5.2% to 27.6%] vs 27.6% [95% CI: 19.5% to 38.0%], *p* < 0.001, although only stage N1 and N3 as per AJCC 7th edition staging, remained associated with higher rates of LRF after adjusting for mrT. The total number of nodes detected was independently associated with LRF (HR 1.13 (95% CI: 1.02 to 1.26], *p* = 0.016). When applying AJCC 8th edition N-stage, we were unable to obtain estimates for 3-year LRF for the N1b and N1c categories due to the few numbers of external iliac nodes detected. We identified an increasing 3-year LRF by nodal location as follows: perirectal without pre-sacral nodes, 23.8%; inguinal nodes, 25.2%; pre-sacral nodes (with or without perirectal nodes), 37.8%; and internal iliac nodes, 33.3% (Table S4).

Both mrEMVI and mrTSH were associated with reduced 3-year LRF (Fig. 2). On adjusting for mrT and LN+, mrTSH retained its association with LRF (HR 2.66 [95% CI: 1.29 to 5.48], *p* = 0.008), whilst mrEMVI did not (HR 2.31 [95% CI: 0.95 to 5.61], *p* = 0.064).

### Comparisons of AJCC 7th and 8th TNM staging systems

The prediction of OS was similar for 7th edition AJCC (C-statistic = 0.641 [95% CI: 0.580 to 0.701]) and 8th edition (C-statistic = 0.673 [95% CI: 0.612 to 0.734]; mean difference = 0.044 [95% CI: −0.053 to 0.120]). LRF by AJCC 7th and AJCC 8th TNM staging demonstrate similar discrimination (7th, C-statistic = 0.693 (95% CI: 0.628 to 0.758), 8th, C-statistic: 0.739 (95% CI: 0.674 to 0.805; mean difference = 0.047 [95% CI: −0.046 to 0.138]).

### Distant metastatic failure

There were 33 (14.5%) DMF events with an actuarial 5-year DMF rate of 15.5% (95% CI: 11.3% to 21.2%). Nodal positivity, mrTSH and mrEMVI, but not mrT-stage or mrT (continuous variable), were independently associated with 5-year DMF, (Table S5, Table S6 and Fig. S8).

## Discussion

### Summary of main findings

Our study is the first sizeable analysis with long-term follow-up to allow estimations of the prognostic significance based on pre-treatment MR imaging parameters on OS and LRF in patients with SCCA. There were four main findings. First, there were strong to perfect agreements within same- and between-institute radiologists for mrT- and mrN- parameters but no agreement for mrEMVI and mrTSH. Second, poor overall survival (OS) was associated with increasing mrT, nodal positivity and mrEMVI. Third, increased loco-regional failure (LRF) was associated with increasing mrT, nodal positivity and mrTSH. Stratification of tumour size at mrT< or ≥4 cm, as is currently being used in the UK PLATO trial, was a clinically relevant stratification cut-off point. Fourth, the discrimination for OS and LRF were similar whether AJCC TNM 7th or 8th editions were used.

### Context of other literature

Our results agree with several studies that have reported prognostic discrimination within the T-staging system only occurs between T1-2 and T3-4 groups [8, 11, 12, 35]. The RTOG 98-11 trial data demonstrated that increasing T-stage was associated with reduced survival when stratified by nodal status, but MR was not mandated [14]. In the AJCC T-staging system, T2 is a broad category ranging from tumour size 2 cm to 5 cm, and is clinically heterogeneous. We reported mrT as a continuous parameter provides more information on risk, and its measurement exhibited good intra- and inter-observer agreement, whilst using 4 cm to separate patients into two T-stage groups provided a clinically relevant cut-point, supporting this stratification in the current PLATO trial.

The AJCC TNM staging system for SCCA was updated from the 7th edition to the 8th edition in 2017 (and again recently updated to 9th edition in 2023 [30]), with the main changes from 7th to 8th editions (and retained in the 9th edition) occurring within the nodal component [25]. The T-staging component has remained unchanged since the 1980s. Studies describing T-staging as an adverse prognosticator have examined the TNM system without addressing other tumour or morphological features, which can now be evaluated using MR [8, 10–12, 14]. Overall, we demonstrate limited discrimination of 8th edition, similar to 7th edition, similar to a recent study [36]. The new AJCC 8th edition nodal staging system proposes a binary nodal category, encompassing all LN+, largely based on the RTOG 98-11 trial data [14]. Studies previously reporting on AJCC 7th edition N-stage stratification demonstrated limited information is gained, with some demonstrating that only the N3 stage provided prognostic information [8, 37], consistent with our findings. There is more emphasis on the presence of external iliac nodes in the AJCC 8th edition N-stage, with the subcategories N1b and N1c representing their presence. However, in this series, we found very few patients with involved external iliac nodes which brings the clinical usefulness of these subcategories into question. Examining individual nodal fields, we found involvement of the pre-sacral group, a subgroup of the perirectal nodes, is consistently associated with poor prognosis and that the total number of nodes detected is independently associated with OS and DMF. New AJCC TNM editions should consider such information.

During this study, the AJCC 9th edition was published and reviewed by Janczewski et al. [30], who pointed out that survival analysis of the AJCC 8th edition ‘revealed a lack of hierarchical order in which stage IIIA anal cancer was associated with a better prognosis than stage IIB disease’. These findings suggest that tumour stage has a greater effect on survival than lymph node status, but as we did not model at the full TNM staging level, the updated staging system does not affect our findings.

EMVI is a known adverse prognosticator in rectal adenocarcinoma, both radiologically and pathologically [38–41]. We reported the first study to characterise EMVI on MR in SCCA. At less than 6%, its proportion on anal cancer appears lower than in rectal cancer [31, 40]. Reasonable agreement of mrEMVI with histology, with a kappa score of 0.64 [31] and a sensitivity and specificity 62% and 88% [40] were seen in rectal studies. In anal cancer, such validation is impossible as surgical resection is not standard care post-CRT. mrEMVI was associated with lower OS and higher rates of LRF and DMF in univariable analyses but only remained associated with OS and DMF after adjusting for mrT and nodal status.

This study also evaluated mrTSH. Tumour signal heterogeneity may be associated with tumour factors such as hypoxia and necrosis. Quantitative methods of tumour heterogeneity evaluation have been reported, for example in 40 patients with SCCA with baseline and post-treatment MR scans, associations were reported between statistical signal heterogeneity parameters (skewness, entropy and energy) and disease recurrence [17].

Over half of the patients in the current series displayed rectal extension of the tumour and a quarter ischio-anal fossa infiltration. These numbers are broadly in line with Goh et al. who described rectal extension in 43% and ischio-anal fossa infiltration in 24% of patients in a 35-patient study [42]. Although the cohort in the present study is much larger, like Goh et al., we found no association of these features with oncological outcomes as well as poor reproducibility.

### Limitations and strengths

This study has a number of limitations. First, this is a re-analysis from a single specialist centre with potential for selection and re- or mis-classification biases. To mitigate against some of these biases, the readers were blinded to outcome during re-characterisation. Second, many MR scans were performed at hospitals other that the cancer centre. However, MR scan quality was high (only three scans excluded) and regional hospital protocols were standardised. Third, there were no routine surgical specimens to validate findings on pathology. Fourth, we did not include diffusion-weighted MR imaging, which may give additional insights for predicting treatment response [43]. Fifth, there were poor intra- and inter-observer agreements for the novel signals mrEMVI and mrTSH. Further training may improve the reproducibility of these parameters.

The study has several strengths. First, the cohort is large with a detailed, systematic morphological characterisation of anal cancer MR cases, using a proforma-based reporting system. Second, a number of radiological features of anal tumours and nodes were examined and related to outcome. Third, intra- and inter-observer variabilities were tested to provide an estimate of internal and external reproducibility.

### Unanswered questions and future research

There is a concern that current risk stratification systems (e.g. AJCC) in SCCA are outdated and may be too crude to provide adequate prognostication for personalised therapy and optimisation of RT planning, and can be refined using MR staging. Alternative strategies to T-staging need to be considered, e.g. employing different stage categories such as <4 cm and ≥4 cm or utilising T size as a continuous variable within multiparametric models. Novel *beyond TNM* tumour features have been identified in this study that may improve risk stratification. Given the differential risk of OS and LRF amongst different nodal groups, the current AJCC versions 8 and 9 binary nodal system may be sub-optimal for clinical treatment planning. Further studies are in progress to externally validate, standardise and optimise the reproducibility of these findings to improve generalisability prior to multi-centre validation with the aim of harnessing the strength of high-resolution MR to enhance prognostication in anal cancer. The ultimate goal is to use such parameters in conjunction with biological prognostic markers such as HPV and p16 status, and measurement of tumour infiltrating lymphocytes within multiparametric models [44].

## Supplementary information

Supplemental Material

## Data Availability

The datasets supporting this article are stored in a secured research database and may be available upon presentation of formal approval.
