# Supplementary material for: Pre-treatment magnetic resonance imaging in anal cancer: large-scale evaluation of mrT, mrN and novel staging parameters
Source: Br J Cancer. 2024 Aug 21;131(7):1137–46. doi: 10.1038/s41416-024-02759-8 (PMC11442706; doi:10.1038/s41416-024-02759-8)
Supplement: Supplementary file 1 — Supplemental Material [file 41416_2024_2759_MOESM1_ESM.docx]

**Electronic Supplementary Material**

**Pre-treatment Magnetic Resonance Imaging in Anal Cancer: large-scale evaluation of mrT, mrN and novel staging parameters**

**Sekhar et al.**

### MRI ACQUISTION PROTOCOL AT THE CHRISTIE NHS FOUNDATION TRUST

Table S1 demonstrates the MRI acquisition protocol employed at The Christie NHS FT. As a substantial proportion of the scans were performed in various other institutions, this acquisition protocol does not apply to all patient scans included in this study. However, quality criteria have been employed to ensure the scans were of diagnostic quality.

Table S1: The MRI acquisition protocol utilised at The Christie NHS FT.

|  | **Sequences** | **Acquisition Parameters** | **Comment** |
| --- | --- | --- | --- |
| Tumour | HR^1^ T2 sagittal TSE | TR/TE 5390/100 ms; NEX 3; ST 3 mm; FOV 200 | Sacral promontory to below the anal margin |
| Tumour | HR^1^ T2 axial TSE | TR/TE 5030/100 ms; NEX 2; ST 3 mm; FOV 200 | Perpendicular to long axis of anal canal, x 2 overlapping blocks |
| Tumour | HR^1^ T2 coronal TSE | TR/TE 6500/137 ms; NEX 2; ST 3 mm; FOV 200 | Parallel to long axis of anal canal |
| Tumour | Diffusion axial T2-weighted SS-EPI | TR/TE 3900/ 89 ms; NEX 5; ST 5 mm; FOV 320 | b values 50,400,800/1000 |
| Tumour | STIR axial  STIR coronal / sagittal | TR/TE 9840/97 ms; NEX 1; ST 3mm  TR/TE 5960/68 ms; NEX 2; ST 3mm | Only if suspicion of fistula |
| Abdomen / Pelvis | T1 axial SE | TR/TE 400/12 ms; NEX 1, ST 5mm; FOV 380 | X 2 overlapping blocks, renal hila to symphysis |
| Abdomen /pelvis | T1 coronal SE | TR/TE 668/19 ms; NEX 2, ST 6mm; FOV 490 | Diaphragm to symphysis pubis |

HR: High Resolution; TSE: Turbo Spin Echo; TR: Repetition Time; TE: Echo Time; NEX: Number of Excitations; ST: Slice Thickness; FOV; Field of View; SS-EPI: Single Shot Echo Planar Imaging; STIR: Short Tau Inversion Recovery Sequence.

### MR Tumour characterisation

Tumours located above the anal verge were classified as canal tumours; those entirely below the verge as margin tumours (Figure S1). The verge was defined as the point at which the squamous mucosa of the distal anal canal joins the hair-bearing perianal skin. Tumours straddling the verge were considered as canal tumours. The anterior-posterior (AP), left-right (LR) and cranio-caudal (CC) size were recorded to determine the longest diameter, which was taken as the mrT (Figure S2). Additionally, we sought to explore whether other stratification of tumour size might be prognostic. We specifically stratified into a binary category of mrT < 4 cm and ≥ 4 cm as used in the currently recruiting large UK PLATO trial and hypothesised to be prognostic for loco-regional failure. This cut-off also approximated to the median mrT size for this cohort.

We classified lymph node involvement if either or both of the following two morphological criteria were met: (i) signal heterogeneity within the node, representative for necrosis; and (ii) border irregularity, representative of extracapsular tumour invasion (Figure S3). Nodal fields were separated into two midline fields: (i) mesorectal and (ii) presacral; and three bilateral fields: (iii) internal iliac; (iv) inguinal; and (v) external iliac. Boundaries were anatomically defined on MR imaging using the Australasian Gastrointestinal Trials Group (AGITG) guidelines (Table S2), similar to the UK National Guidance for IMRT in anal cancer. We included obturator nodes in the internal iliac region, as the obturator region number of cases is too small for separate analysis. The common iliac region was not evaluated as it was not routinely included in all studied MR scans. Extension into the rectum was documented if the tumour extended superiorly beyond the ano-rectal junction, radiologically identified by the puborectalis sling. From the above T- and N- features, we derived the stage according to AJCC 7^th^ and 8^th^ edition (Table S3).

Other tumour characteristics identified were: (i) MR defined extramural vascular invasion (mrEMVI, illustrated in Figure S4), identified as tubular/serpiginous extension of tumour signal along the line of a vascular structure; (ii) MR defined tumour signal heterogeneity (mrTSH, illustrated in Figure S5), identified as areas of mixed signal intensity in the primary tumour on high resolution T2-weighted images; (iii) sphincter infiltration (illustrated in Figure S6), categorised as confined to the internal sphincter, confined to the external sphincter, or having breached the external sphincter; (iv) sepsis (illustrated in Figure S7), defined as the presence of any tumour associated abscess or fistula; and (v) organ extension, defined as invasion into adjacent organs, such as vagina, urethra and bladder (T4 tumour).

### MR PICTORIAL EXAMPLES

***Tumour Position***

The anal verge was defined as the point at which the squamous mucosa of the distal anal canal joins the hair-bearing perianal skin. Radiologically the difference was identified by a change of signal intensity between the canal and margin, which is usually in line with the inferior most fibres of the external sphincter. The dentate line was taken to be at the junction between the upper and middle third of the anal canal. Extension of the tumour into the rectum was radiologically identified by extension above the puborectalis sling.high-resolution T2-weighted in the coronal plane. Red lines mark the level of the anorectal junction; dashed white lines the level of the dentate line; solid white vertical lines the length of the anal canal and red arrows the tumour. Top row demonstrates tumour positions in relation to canal and margin tumours: A) Tumour seen entirely within the anal canal, extending from just below the anorectal junction and reaching the dentate line; B) Tumour seen straddling the verge, with both anal canal and anal margin components; C) Tumour seen limited to the anal margin area. Bottom row demonstrates tumour position in relation to the dentate line: D) Tumour seen below the dentate line; E) Tumour seen straddling the dentate line and also extending above the anorectal junction into the low part of the rectum; F) Tumour localised entirely above the dentate line without rectal extension.

The dentate line is an imaginary line (which cant actually see it on MR) at the junction of upper third and lower two third of the anal canal. So on images, the position of this white dashed line should be consistent with respect to the length of anal canal shown, so for example in Figure S1b it is drawn at the half way mark; Figure S1d, it is shown at junction of upper third and lower two thirds like it should be.

*
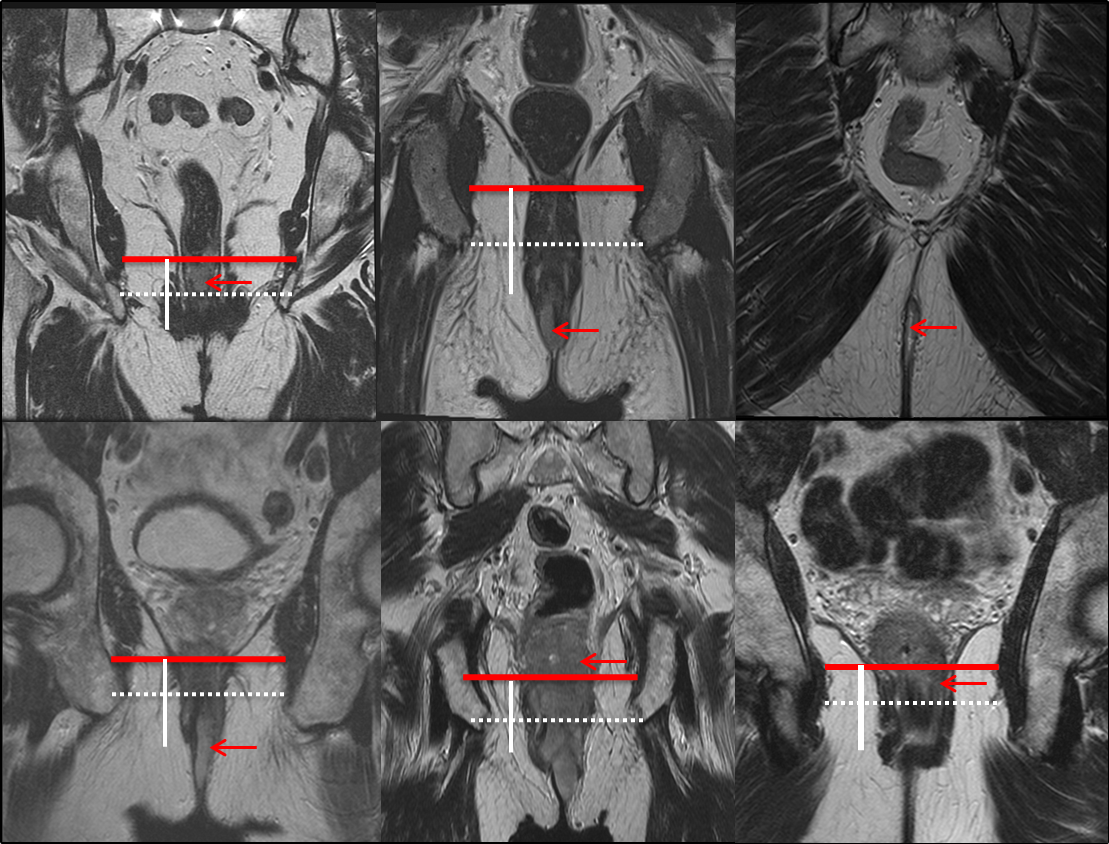
*

**C**

**A**

**B**

**E**

**F**

**D**

Figure S1: Examples of tumour position demonstrated on high-resolution T2-weighted MRI in the coronal plane. Red lines mark the level of the anorectal junction; dashed white lines the level of the dentate line; solid white vertical lines the length of the anal canal and red arrows the tumour. Top row demonstrates tumour positions in relation to canal and margin tumours: A) tumour seen entirely within the anal canal, extending from just below the anorectal junction and reaching the dentate line; B) tumour seen straddling the verge, with both anal canal and anal margin components; C) tumour seen limited to the anal margin area. Bottom row demonstrates tumour position in relation to the dentate line: D) tumour seen below the dentate line; E) tumour seen straddling the dentate line and also extending above the anorectal junction into the low part of the rectum; F) tumour localised entirely above the dentate line with extension into the lower rectum.

***T-Stage***

The anterior-posterior (AP), left-right (LR) and cranio-caudal (CC) diameter sizes were recorded to determine the largest diameter. The AP and LR diameters were recorded on the axial plane at the point of maximal dimension. The longest diameter was noted, and the next dimension taken at an axis perpendicular to this. CC dimension was measured in either the coronal or sagittal plane. The largest measurement was taken as the mrT-size and derived mrT-stage as per AJCC 7^th^ edition staging.

**I**

**F**

**C**

**H**

**E**

**B**

**G**

**D**

**A**


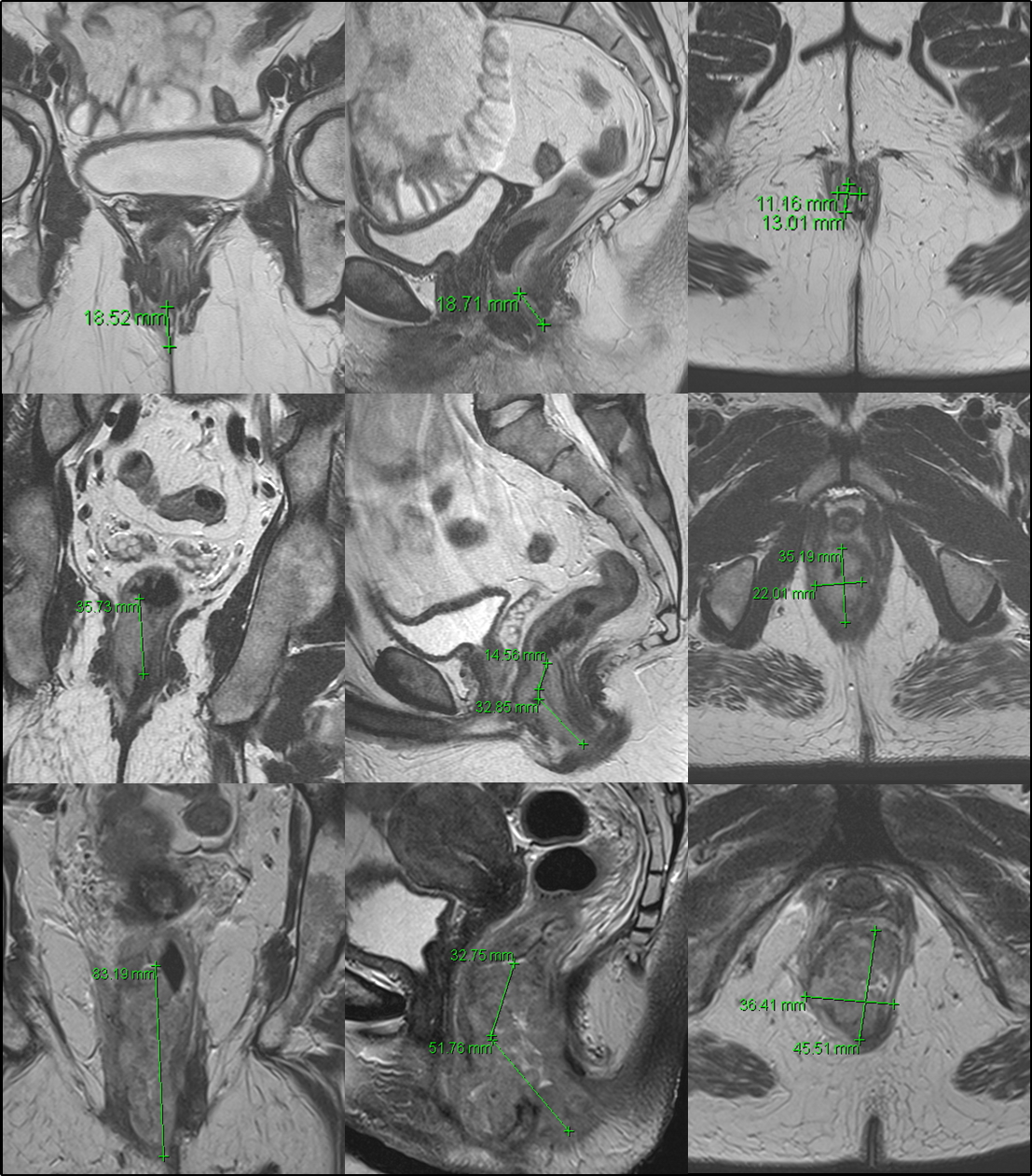


Figure S2: Examples of tumour stage (AJCC 7^th^ Edition) demonstrated on high-resolution T2-weighted MRI, with the green callipers denoting cross-sectional size. Top row demonstrates a T1-stage tumour on; A) a coronal view with a cranio-caudal [CC] size of 1·9cm; B) the sagittal view corroborates the CC size to be of 1·9 cm and C) the axial view demonstrates anterior-posterior [AP] x left-right [LR] dimensions of 1·3 cm x 1·2 cm, respectively. Middle row demonstrates a T2 tumour on D) a coronal view with a tumour CC size of 3·6 cm with the tumour extending into the lower rectum (the red line indicates correct measurement); E) a sagittal view demonstrating a tumour CC size of 4·8 cm highlighting importance of correlation in all three planes to get the maximum dimension and F) an axial view demonstrates AP x LR dimensions of 3·5 cm x 2·2 cm respectively. Bottom row depicts a T3 tumour on G) a coronal view demonstrating tumour CC size of 8·3 cm with the tumour extending into the rectum; H) a sagittal view demonstrating tumour CC size of 8·5 cm and I) an axial view demonstrating a tumour AP size of 4·6 cm and an LR size of 3·6 cm.high-resolution T2-weighted, with the green callipers denoting cross-sectional size. Top row demonstrates a T1-stage tumour on; A) a coronal view with a cranio-caudal [CC] size of 1.9 cm; B) a sagittal view corroborates the CC size to be of 1.9 cm and C) the axial view demonstrates anterior-posterior [AP] x left-right [LR] dimensions of 1.3 cm x 1.2 cm, respectively. Middle row demonstrates a T2 tumour on D) a coronal view with a tumour CC size of 3.6 cm with the tumour extending into the lower rectum; E) a sagittal view demonstrating a tumour CC size of 4.9 cm highlighting importance of correlation in all three planes to get the maximum dimension and F) an axial view demonstrates AP x LR dimensions of 3.5 cm x 2.2 cm respectively. Bottom row depicts a T3 tumour on; G) a coronal view demonstrating tumour CC size of 8.3 cm with the tumour extending into the rectum; H) a sagittal view demonstrating tumour CC size of 8.5 cm and I) an axial view demonstrating a tumour AP size of 4.6 cm and an LR size of 3.6 cm.

### MRI NODAL CHARACTERISATION – PICTORIAL EXAMPLES


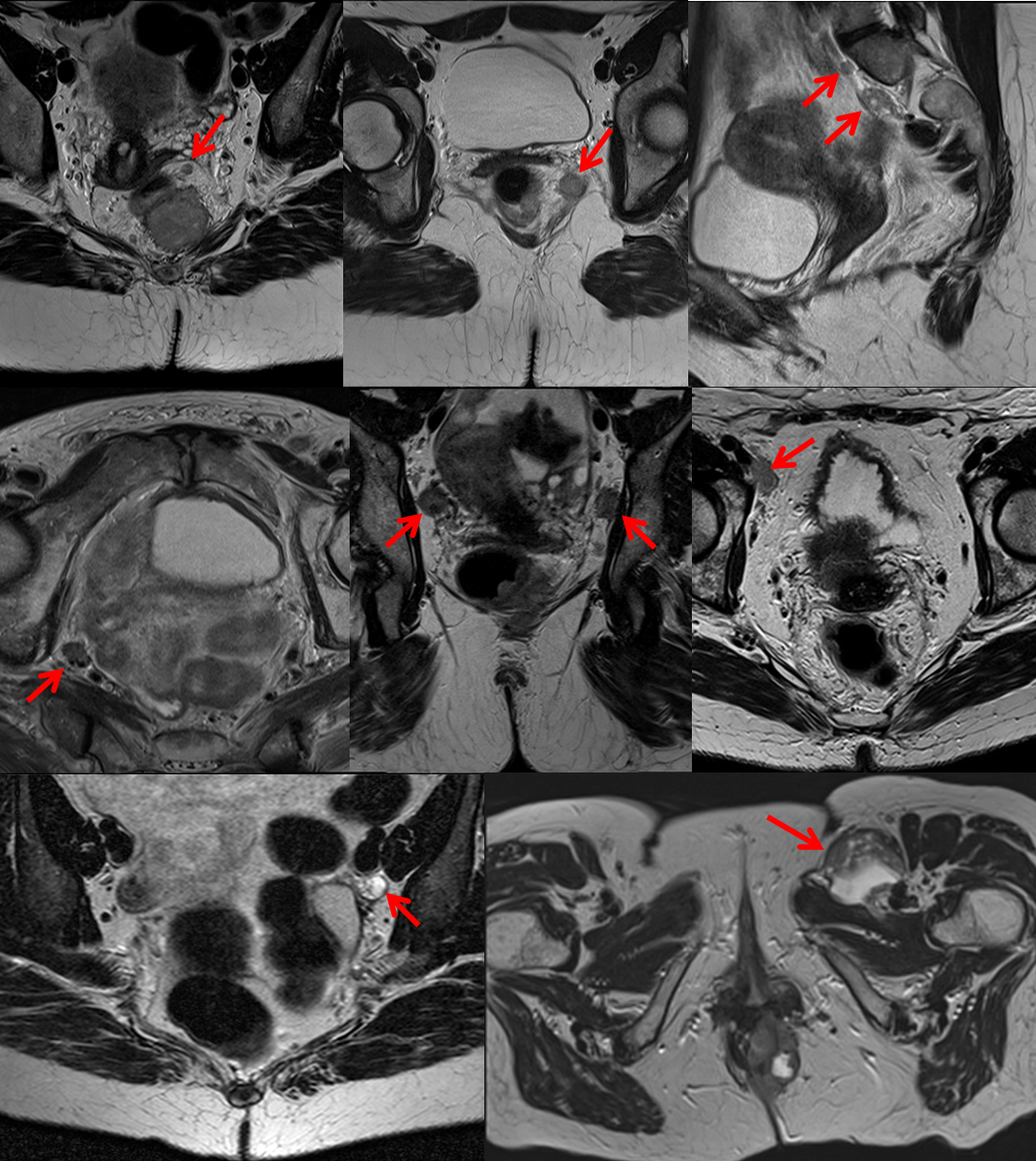


**H**

**G**

**F**

**E**

**D**

**C**

**B**

**A**

**E**

**D**

**Figure S3: Examples of morphological criteria of lymph nodes, marked with red arrows, demonstrated on T2-weighted high-resolution MRI (unless stated otherwise):, marked with red arrows, A) MR image in axial plane with a mesorectal node at the 12 o’clock position, demonstrating signal heterogeneity; B) MR images in axial plane with a mesorectal node at the 3 o’clock position, displaying signal heterogeneity and an irregular outline, involving the mesorectal fascia; C) MR images in sagittal plane with pre-sacral nodes at the L5 and lower border of S1 level, displaying signal heterogeneity; D) MR image in axial plane with right internal iliac node lying outside of the mesorectal fascia displaying signal heterogeneity; E) MR image in coronal plane with bilateral internal iliac nodes with signal heterogeneity and an irregular outline. The node on the left also shows extracapsular extension and possible early infiltration of the left obturator internus muscle; F) right external iliac node displaying signal heterogeneity and an irregular outline adjacent to the external iliac vessels; G) a necrotic left external iliac node; and H) large field of view T2-weighted scan demonstrating grossly enlarged left inguinal node with signal heterogeneity, necrosis and an irregular border with extracapsular spread into the adjacent adductor muscle.**

### CRITERIA FOR NODAL FIELD BOUNDARIES

Table S2: Anatomical boundaries of nodal fields modified from the Australasian Gastrointenstinal Trials Group criteria.

|  | **Cranial** | **Caudal** | **Lateral** | **Medial** | **Anterior** | **Posterior** |
| --- | --- | --- | --- | --- | --- | --- |
| **Internal Iliac** | Bifurcation of the CIA – usually corresponds to the L5-S1 interspace. | Where the fibres of the levator ani insert into the obturator fascia and obturator internus, and can be demarcated either at the level of the obturator canal, or at the level where there is no space between the obturator internus muscle and the midline organs. | The medial edge of the obturator internus muscle (or bone where muscle is not present) in the lower pelvis, iliopsoas in the upper pelvis. | The mesorectum and the presacral space in the lower pelvis. In the upper pelvis, a 7 mm medial margin is recommended from the internal iliac vessels | The obturator internus muscle or bone in the lower pelvis. In the upper pelvis, a 7 mm margin around the internal iliac vessels. | The bony pelvis |
| **External Iliac** | Bifurcation of the CIA. | The level where the external iliac vessels are still located within the bony pelvis before continuing as the femoral artery. This transition usually occurs between the acetabulum’s roof and the superior pubic rami. | The iliopsoas muscle. | Usually, the bladder forms the medial wall; otherwise a 7 mm margin around the vessels. | A 7 mm margin anterior to the external iliac vessels. | The internal iliac lymph node group. |
| **Inguinal** | The level where the external iliac artery leaves bony pelvis to become the femoral artery. | At the point where the muscles of the sartorius and adductor longus cross OR at the inferior slice demonstrating the lesser trochanter. | The medial edge of sartorius or ilio-psoas. | A 10 to 20 mm margin around the femoral vessels. The medial third to half of the pectineus or adductor longus muscle serves as an approximate border. | A minimum of 20 mm margin on the inguinal vessels. | The bed of the femoral triangle is formed by the iliopsoas, pectineus and adductor longus muscles. |
| **Mesorectal** | The level of the recto-sigmoid junction; best identified where the rectum runs anteriorly to join the sigmoid colon. | The ano-rectal junction, defined by where the levator muscles fuse with the external sphincter muscles, where mesorectal fat/space is no longer seen tapering inferiorly. Often this level can be identified by a plane drawn from the caudal edge of the pubic symphysis to the Coccyx transecting the rectum. | In the lower pelvis, the border is the medial edge of the levator ani. In the upper pelvis, it is the internal iliac lymph node group and the medial edges of the mesorectal fascia and levator ani. |  | Males: the boundary is formed by the penile bulb and prostate in the lower pelvis, and by the posterior edge of the seminal vesicles and bladder in the mid pelvis.  Females: boundary is formed by the bladder, vagina, cervix, and uterus. | The presacral space. |
| **Pre-sacral** | The sacral promontory, defined at the L5-S1 interspace. | The inferior edge of the coccyx. | The SIJs. |  | 10 mm anterior to the anterior sacral border encompassing any lymph nodes or presacral vessels. | The position of the anterior border of the sacral bone. |
| **Common Iliac** | Aortic bifurcation. | Common iliac bifurcation. | Mesocolon. | Sacrum. | Psoas muscles. |  |

SIJ: Sacro-iliac Joint; CIA: Common Iliac Artery

The obturator volume refers to an area containing the nodes that are associated with the obturator artery, a branch of the internal iliac artery and are found in relation to the internal foramen of the obturator canal. However, for clarification, the nodes (also called obturator nodes), which are located in a quadrangular area bounded superiorly by the external iliac vein, posteriorly by the internal iliac artery and inferiorly the obturator nerve, form part of the medial group of external iliac lymph nodes and will thus be considered to be within the external iliac nodal region.

### AJCC 7^th^ and 8^th^ EDITION STAGING FOR ANAL CANCER

Table S3: AJCC 7^th^ and 8^th^ edition staging for anal cancer

| T Stage | | | |
| --- | --- | --- | --- |
| Tx | Primary tumour cannot be assessed | | |
| **T0** | No evidence of primary tumour | | |
| **T1** | Tumour 2 cm or less in greatest dimension | | |
| **T2** | Tumour more than 2 cm but not more than 5 cm in greatest dimension | | |
| **T3** | Tumour more than 5 cm in greatest dimension | | |
| **T4** | Tumour of any size invades adjacent organ(s), e.g., vagina, uterus, bladder | | |
| N Stage AJCC 7^th^ Edition | | N Stage AJCC 8^th^ Edition | |
| **Nx** | Regional lymph nodes cannot be assessed | Nx | Regional lymph nodes cannot be assessed |
| **N0** | No regional lymph node metastasis | N0 | No regional lymph node metastasis |
| **N1** | Metastasis in perirectal lymph node(s) | N1a | Metastasis in inguinal, mesorectal, or internal iliac lymph nodes |
| **N2** | Metastasis in unilateral internal iliac and/or inguinal lymph node(s) | N1b | Metastasis in external iliac lymph nodes |
| **N3** | Metastasis in perirectal and inguinal lymph nodes and/or bilateral internal iliac and/or inguinal lymph nodes | N1c | Metastasis in external iliac with any N1a nodes |
| M stage | | | |
| **M0** | No distant metastasis | | |
| **M1** | Distant metastasis | | |

The AJCC 8^th^ edition Cancer Staging Manual was published in 2018. Although the T-stage parameters remain unchanged, N-staging has undergone a dramatic revision having been condensed into a binary system of any node positivity versus node negativity, with subgroups embedded within dependent on the external iliac nodal field, a nodal field that was previously considered to constitute metastatic (M1) disease in the 7^th^ edition staging system.

### MRI ADDITIONAL TUMOUR CHARACTERISATION – PICTORIAL EXAMPLES

***Extra-Mural Vascular Invasion***

***
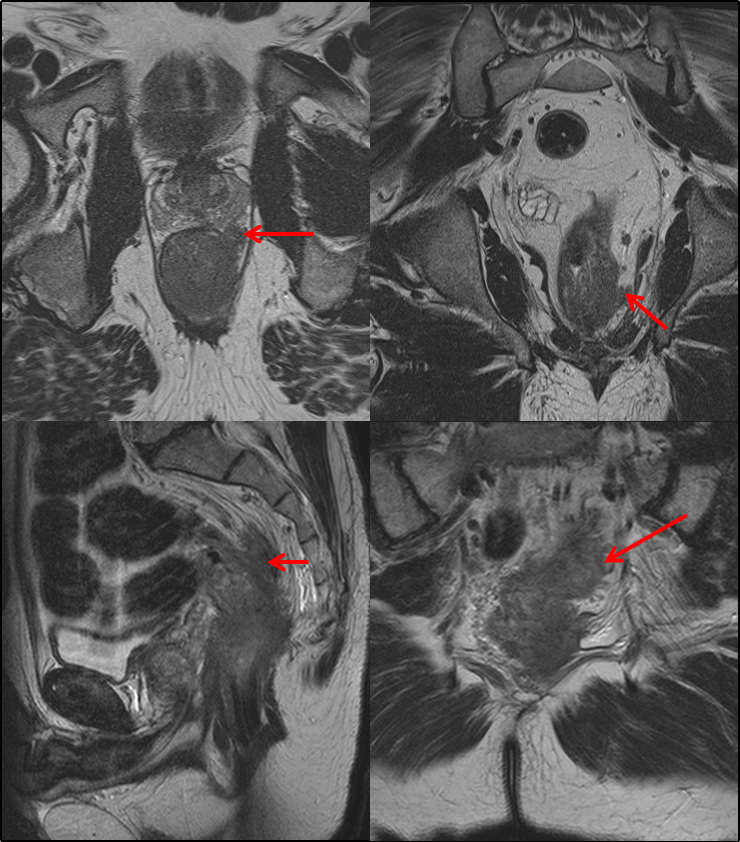
***

**A**

**B**

**C**

**D**

**Figure S4: Examples of tumour extra-mural vascular invasion (EMVI), marked with a red arrow, demonstrated on high-resolution T2-weighted MRI. Top row demonstrates early EMVI in A) an axial view with EMVI seen at the 1 o'clock position and B) a coronal view of the same tumour with EMVI seen as a subtle plaque of tumour signal extending along the vessels. Bottom row demonstrates advanced EMVI in C) a sagittal view with a thick tubular plaque of tumour signal seen extending posterior to the primary tumour and D) the same is also seen in the coronal view extending superior to the tumour along the course of vessels.. Top row demonstrates early EMVI in A) an axial view with EMVI seen at the 1 O'clock position and B) a coronal view of the same tumour with EMVI seen as a subtle plaque of tumour signal extending along the vessels. Bottom row demonstrates advanced EMVI in C) a sagittal view with a thick tubular plaque of tumour signal seen extending posterior to the primary tumour and D) the same is also seen in the coronal view extending superior to the tumour along the course of vessels.**

***Tumour Signal Heterogeneity***

***
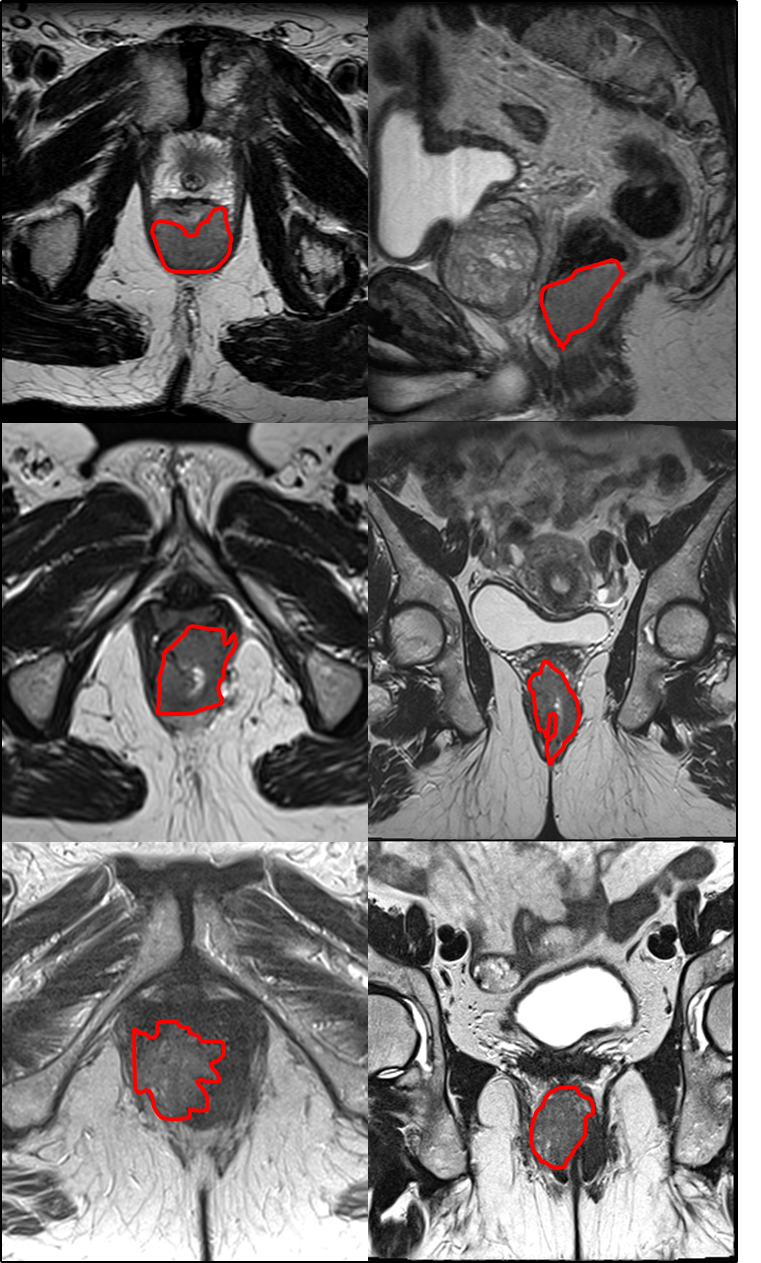
***

**A**

**B**

**C**

**D**

**E**

**F**

**Figure S5: Examples of tumour signal heterogeneity demonstrated on high-resolution T2-weighted MRI with the tumours contoured in red. Top row demonstrates in A) axial and B) sagittal planes, a 5·5 cm sized anal canal tumour with a homogeneous intermediate signal and no tumour heterogeneity evident and no evidence to suggest necrosis or ulceration. Middle row images demonstrate in C) axial and D) coronal planes, an anal canal tumour exhibiting intermediate signal intensity and high signal intensity with a central area of necrosis. Bottom row demonstrates in E) axial and F) sagittal planes, an anal canal tumour demonstrating**

**generally heterogeneous intermediate signal intensity with speckled areas of high signal within the tumour.*Extent of Sphincter Infiltration***

*
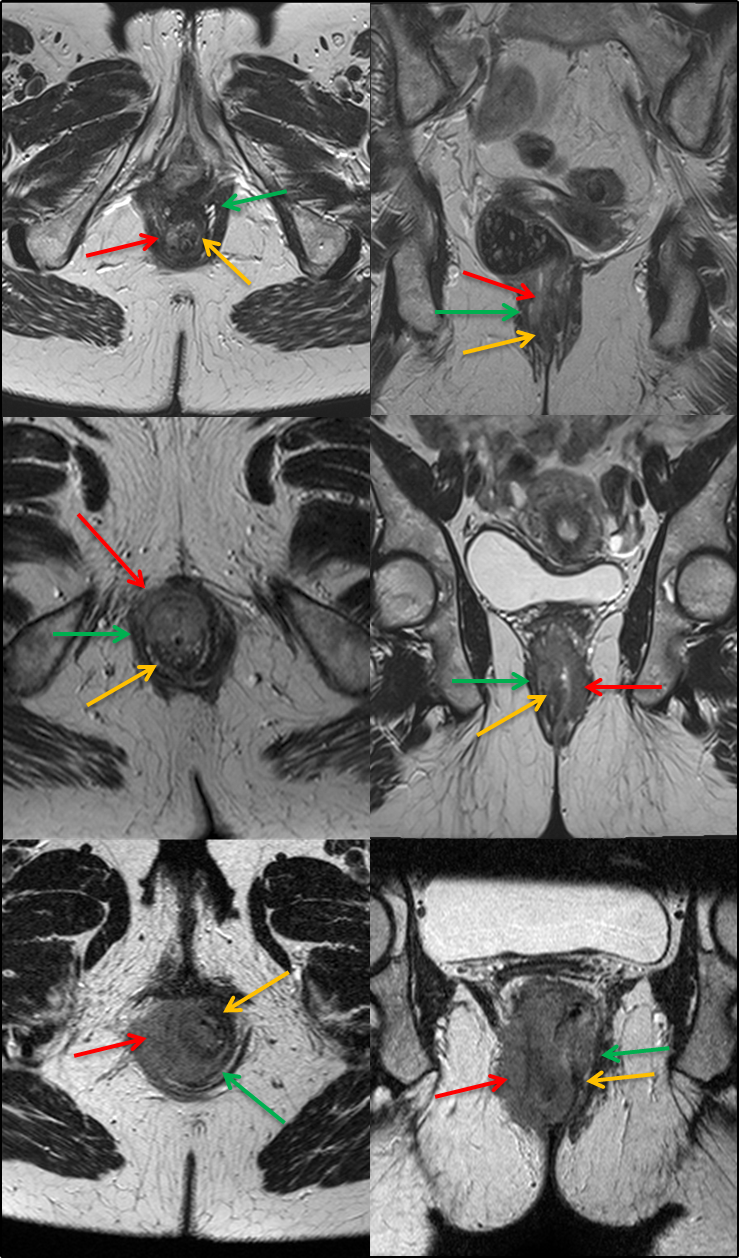
*

**A**

**B**

**C**

**D**

**E**

**F**

Figure S6: Examples of tumour infiltration through the anal sphincters demonstrated on high-resolution T2-weighted MRI; tumour is marked with a red arrow, the internal sphincter with an orange arrow and the external sphincter with a green arrow. Top row demonstrates in A) axial and B) coronal planes an anal canal tumour contained within the internal sphincter. Middle row demonstrates in C) axial and D) coronal planes an anal canal tumour extending into the external sphincter, but not beyond it. Bottom row demonstrates in E) axial and F) coronal planes a large anal tumour with trans-sphincteric extension infiltrating through both internal and external sphincters into the adjacent ischio-anal fat.high resolution T2 weighted highhi; tumour is marked with a red arrow, the internal sphincter with an orange arrow and the external sphincter with a green arrow. Top row demonstrates, in axial A) and coronal B) planes an anal canal tumour contained within the internal sphincter. Middle row demonstrates in axial C) and coronal D) planes an anal canal tumour extending into the external sphincter, but not beyond it. Bottom row demonstrates in axial E) and coronal F) planes a large anal tumour with trans-sphincteric extension; infiltrating through both internal and external sphincters into the adjacent ischio-anal fat.

**F**

**E**

**D**

**C**

**B**

**A**

***Anal Fistulae and Abscesses***

***
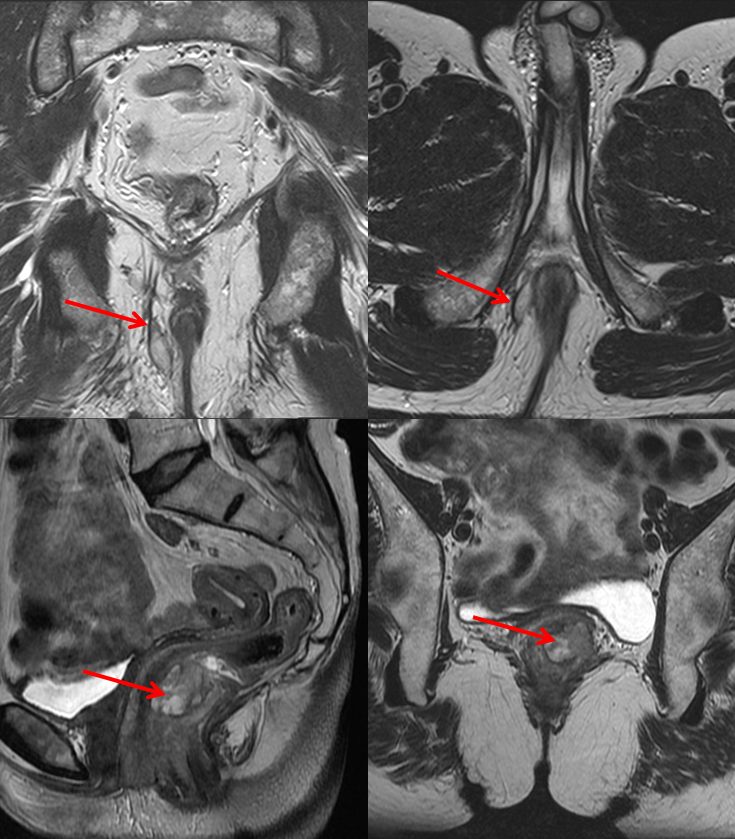
***

**A**

**B**

**C**

**D**

Figure S7: Examples of tumour associated signs of sepsis demonstrated on high-resolution T2-weighted MRI. Top row demonstrates, in A) coronal and B) axial planes, an extra-sphincteric fistulous track seen in the right ischio-anal fossa extending inferiorly towards the anal verge, marked by a red arrow. Bottom row demonstrates, in C) sagittal and D) coronal plane, a large anorectal mass with internal heterogeneous T2 high-signal intensity (red arrow) that is well demarcated and subsequently confirmed as an intra-tumour abscess.high-resolution T2-weighted hihg. Top row demonstrates, in coronal A) and sagittal plane B), an extra-sphincteric fistulous track seen in the right ischio-anal fossa extending inferiorly towards the anal verge, marked by red arrow. Bottom row demonstrates, in sagittal plane C) and coronal plane D), a large anorectal mass with internal heterogeneous T2 high signal intensity (red arrow) that is well demarcated and subsequently confirmed as an intra-tumour abscess.

**C**


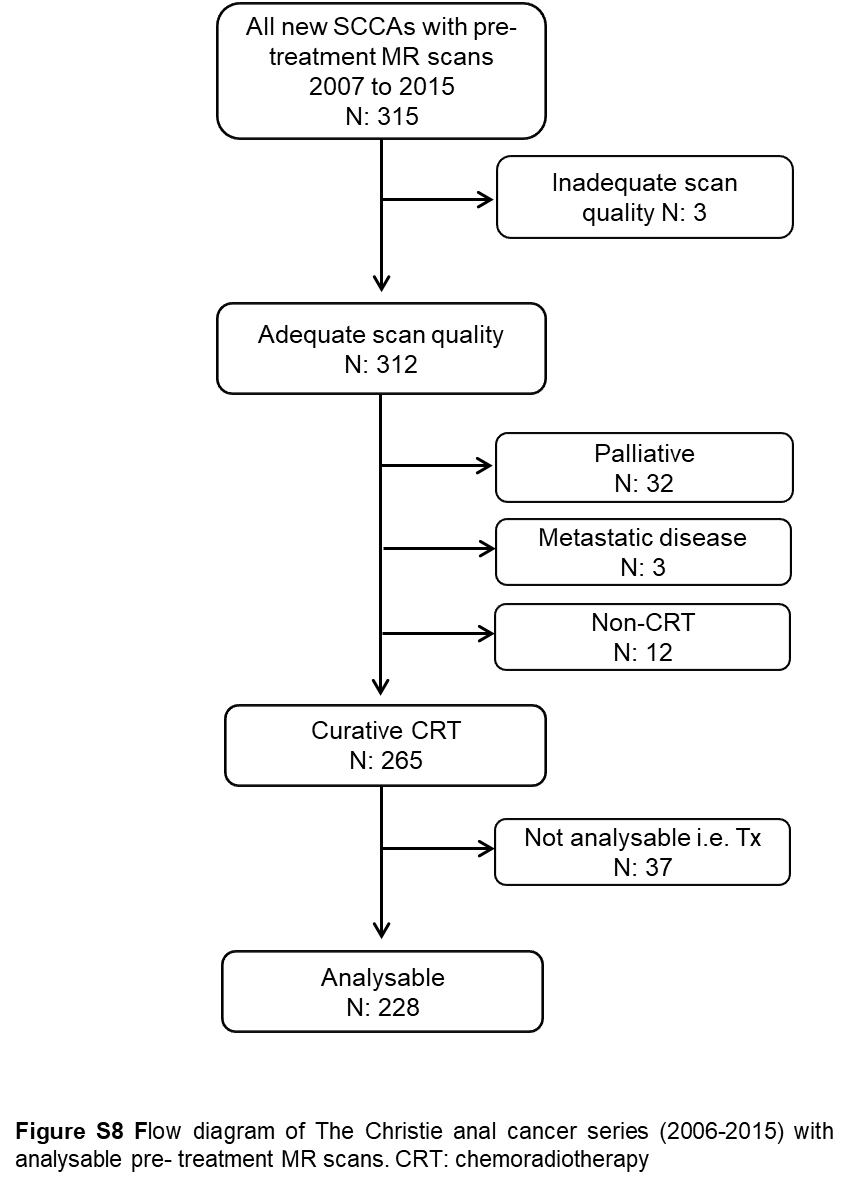


### ASSOCIATIONS OF TUMOUR AND NODAL CHARACTERISTICS WITH TUMOUR SIZE

Table S3: Association of studied MRI derived tumour and nodal parameters with mrT-size in the 265 patient in prognostic analyses, treated with chemoradiotherapy with curative intent.

| **Variable** | **T Size (median, IQR)** | ***p* value** |
| --- | --- | --- |
| **Position** | | |
| **Canal (208)** | 4·35 (3·4 – 5·7) | 0·0002**†** |
| **Margin (20)** | 2·75 (2·3 – 3·6) |  |
| **Rectum Extension** | | |
| **No (88)** | 3·4 (2·5 – 4·5) | <0·0001**†** |
| **Yes (140)** | 4·9 (3·6 – 6·1) |  |
| **Dentate Position** | | |
| **Above (36)** | 3·4 (2·8 – 4·5) |  |
| **Straddling (140)** | 4·8 (3·8 – 6·2) |  |
| **Below (52)** | 3·2 (2·4 – 4·2) | 0·0001‡ |
| **Primary Tumour Heterogeneity** | | |
| **No (111)** | 4 (2·9 – 5·3) | 0·030**†** |
| **Yes (117)** | 4·5 (3·4 – 5·9) |  |
| **Primary Tumour EMVI** | | |
| **No (215)** | 4·2 (3·1 – 5·5) |  |
| **Yes (13)** | 6·1 (5·7 – 6·5) | 0·0004**†** |
| **Primary Sepsis** | | |
| **No (209)** | 4·2 (3·3 – 5·5) |  |
| **Yes (19)** | 4·3 (2·5 – 6·1) | 0·51**†** |
| **Sphincter*** | | |
| **Internal (62)** | 3·4 (2·5 – 4·2) | <0·0001‡ |
| **External (113)** | 4·3 (3·4 – 5·5) |  |
| **Ischio-rectal fossa (51)** | 6·1 (4·4 – 6·7) |  |
| **Primary Organ Extension** | | |
| **No (183)** | 3·9 (3 – 5·1) | <0·0001**†** |
| **Yes (45)** | 5·9 (4·5 – 6·6) |  |
| **Any Nodes** | | |
| **No (136)** | 3·8 (2·9 – 5·1) |  |
| **Yes (92)** | 4·9 (3·6 – 6·1) | <0·0001**†** |
| **Perirectal Nodes** | | |
| **No (166)** | 3·9 (3 – 5·1) |  |
| **Yes (62)** | 5·1 (4 – 6·1) | <0·0003**†** |
| **Sacral** | | |
| **No (204)** | 4·1 (3·1 – 5·3) |  |
| **Yes (24)** | 6·0 (4·4 – 6·6) | 0·0009**†** |
| **Inguinal Nodes** | | |
| **No (195)** | 4·1 (3·1 – 5·6) |  |
| **Yes (33)** | 4·8 (3·9 – 5·5) | 0·05**†** |
| **Internal Nodes** | | |
| **No (210)** | 4·2 (3·1 – 5·5) |  |
| **Yes (18)** | 5·3 (4·2 – 6·1) | 0·002**†** |
| **External Nodes** | | |
| **No (223)** | 4·2 (3·1 – 5·5) |  |
| **Yes (5)** | 5·5 (4·8 – 6·1) | 0·11**†** |
| **Total Nodes** | r=0·2538 | <0·0001§ |

IQR: Interquartile Range

*†Out of 226 assessable tumours (parameter not assessable in two patients);

**†**Wilcoxon-Rank Sum Test; ‡Kruskall-Wallis Test; §Pearson’s Correlation Coefficient;

Values in parentheses represent the number of patients.

**ASSOCIATIONS OF POSITION OF NODAL INVOLVEMENT WITH OVERALL SURVIVAL AND LOCOREGIONAL FAILURE**

Table S4: Association of studied MRI derived position of nodal involvement with overall survival and locoregional failure

| .**Variable** | **5yr OS**  **(95% CI)** | **HR***  **(95% CI)** | ***p* value** | **HR†**  **(95% CI)** | ***p* value** | **3yr LRF**  **(95% CI)** | **HR***  **(95% CI)** | ***p* value** | **HR†**  **(95% CI)** | ***p* value** |
| --- | --- | --- | --- | --- | --- | --- | --- | --- | --- | --- |
| **Perirectal Nodes (198)** | | | | | | | | | | |
| **Negative (136)** | 82·2  (74·5 – 87·6) | Referent |  | Referent |  | 9·0  (5·2 – 15·4) | Referent |  | Referent |  |
| **Positive (62)** | 62·1  (48·7 – 73·0) | 2·40  (1·35 – 4·25) | 0·003 | 2·13  (1·20 – 3·78) | 0·010 | 29·2  (19·5 – 42·3) | 3·59  (1·77 – 7·27) | <0·001 | 2·93  (1·44 – 5·94) | 0·003 |
| **Perirectal Nodes (198)** | | | | | | | | | | |
| **Negative (136)** | 82·1  (74·5 – 87·6) | Referent |  | Referent |  | 9·0  (5·2 – 15·4) | Referent |  | Referent |  |
| **Mesorectal without pre-sacral (38)** | 70·1  (52·5 – 82·2) | 1·80  (0·88 – 3·67) | 0·107 | 1·72  (0·84 – 3·51) | 0·138 | 23·8  (13·1 – 40·7) | 3·02  (1·32 – 6·89) | 0·009 | 2·80  (1·22 – 6·41) | 0·015 |
| **Sacral w/wo PR (24)** | 50·0  (29·1 – 67·8) | 3·46  (1·73 – 6·94) | <0·001 | 2·75  (1·36 – 5·57) | 0·005 | 37·8  (21·7 – 60·1) | 4·54  (1·93 – 10·64) | <0·001 | 3·09  (1·32 – 7·26) | 0·010 |
| **Internal Nodes (154)** | | | | | | | | | | |
| **Negative (136)** | 82·1  (74·5 – 87·6) | Referent |  | Referent |  | 9·0  (5·2 – 15·4) | Referent |  | Referent |  |
| **Positive (18)** | 44·4  (21·6 – 65·1) | 3·79  (1·81 – 7·95) | <0·001 | 3·15  (1·50 – 6·63) | 0·003 | 33·3  (16·6 – 59·7) | 4·02  (1·52 – 10·60) | 0·005 | 3·22  (1·22 – 8·49) | 0·018 |
| **Inguinal Nodes (169)** | | | | | | | | | | |
| **Negative (136)** | 82·1  (74·5 – 87·6) | Referent |  | Referent |  | 9·0  (5·2 – 15·4) | Referent |  | Referent |  |
| **Positive (33)** | 57·6  (39·1 – 72·3) | 2·75  (1·42 – 5·32) | 0·003 | 2·36  (1·21 – 4·61) | 0·012 | 25·2  (13·4 – 44·1) | 3·18  (1·36 – 7·46) | 0·008 | 2·34  (0·99 – 5·57) | 0·054 |

OS: Overall Survival; LRF: Locoregional Failure; HR: hazard ratio; CI: confidence interval. Values in parentheses represent patient numbers;

*Univariable Cox regression; †Multivariable Cox regression adjusted for mrT-size; §Involved field compared with those patients deemed node negative.

### ASSOCIATIONS OF TUMOUR AND NODAL CHARACTERISTICS WITH DISTANT METASTATIC FAILURE

Table S5: Association of studied MRI derived tumour parameters with distant metastatic failure in the 265 patient in prognostic analyses, treated with chemoradiotherapy with curative intent.

| **Variable** | **5yr DMF**  **(95% CI)** | **HR***  **(95% CI)** | ***p* value** | **HR†**  **(95% CI)** | ***p* value** |
| --- | --- | --- | --- | --- | --- |
| **T Stage** | | | | | |
| **T1 (7)** | 0§ | 0§ | 1·0 | 0§ | 1·0 |
| **T2 (124)** | 10·3  (6·0 – 17·5) | Referent |  | Referent |  |
| **T3 (52)** | 24·2  (14·5 – 38·7) | 2·82  (1·27 – 6·29) | 0·011 | 2·45  (1·09 – 5·52) | 0·031 |
| **T4 (45)** | 23·8  (13·1 – 40·9) | 2·39  (1·00 – 5·67) | 0·049 | 1·90  (0·78 – 4·64) | 0·158 |
| **Size (cm) (265)** |  | 1·20 (1·03 – 1·40) | 0·016 | 1·17  (0·99 – 1·38) | 0·058 |
| **Position** | | | | | |
| **Canal (208)** | 17·1  (12·5 – 23·2) | Referent |  | Referent |  |
| **Margin (20)** | 0§ | 0§ | 1·0 | 0§ | 1·0 |
| **Rectal Extension** | | | | | |
| **No (88)** | 8·6  (4·2 – 17·2) | Referent |  | Referent |  |
| **Yes (140)** | 19·9  (14·0 – 27·9) | 2·62  (1·14 – 6·03) | 0·024 | 2·01  (0·86 – 4·69) | 0·11 |
| **Dentate – Position** | | | | | |
| **Above (36)** | 14·3  (6·2 – 31·0) | Referent |  | Referent |  |
| **Straddling (140)** | 19·3  (13·5 – 27·3) | 1·43  (0·55 – 3·73) | 0·468 | 1·27  (0·46 – 3·45) | 0·646 |
| **Below (52)** | 6·4  (2·1 – 18·5) | 0·41  (0·10 – 1·72) | 0·222 | 0·52  (0·12 – 2·20) | 0·373 |
| **Tumour Heterogeneity** | | | | | |
| **No (111)** | 9·7  (5·3 – 17·3) | Referent |  |  |  |
| **Yes (117)** | 21·1  (14·5 – 30·1) | 2·44  (1·26 – 5·13) | 0·019 | 2·15  (1·02 – 4·54) | 0·045 |
| **Primary Sepsis** | | | | | |
| **No (209)** | 16·9  (12·3 – 23·0) | Referent |  | Referent |  |
| **Yes (19)** | 0 | 0§ | 1·0 | 0§ | 1·0 |
| **Primary EMVI** | | | | | |
| **No (215)** | 13·4  (9·4 – 19·0) | Referent |  | Referent |  |
| **Yes (13)** | 56·3  (29·7 – 85·6) | 5·66  (2·32 – 13·81) | <0·001 | 3·95  (1·56 – 9·98) | 0·004 |
| **Sphincter Infiltration**‡ | | | | | |
| **Internal (62)** | 10·1  (4·7 – 21·2) | Referent |  | Referent |  |
| **External (113)** | 18·9  (12·6 – 27·8) | 1·89  (0·76 – 4·70) | 0·172 | 1·28  (0·50 – 3·31) | 0·607 |
| **Ischio-rectal fossa (51)** | 15·6  (7·7 – 29·9) | 1·46  (0·49 – 4·34) | 0·497 | 0·76  (0·23 – 2·51) | 0·658 |
| **Primary Organ Extension** | | | | | |
| **No (220)** | 13·8  (9·5 – 19·9) | Referent |  | Referent |  |
| **Yes (45)** | 23·8  (13·1 – 40·9) | 1·69  (0·78 – 3·63) | 0·182 | 1·15  (0·52 – 2·54) | 0·737 |

DMF: Distant Metastatic Failure; HR: Hazard Ratio; CI: Confidence Interval; Values in parentheses represent number of patients;

*Univariable Cox regression; †Multivariable Cox regression adjusted for T-size and nodal status (T-stage and T-size only adjusted for nodal status);

‡ parameter not assessable in Tx tumours and another two patients, therefore analysis in 226 patients, §: no events occurred.

Table S6: Association of MRI derived nodal field involvement with distant metastatic failure.

| **Variable** | **5yr DMF**  **(95% CI)** | **HR***  **(95% CI)** | ***p* value** | **HR†**  **(adjusted)** | ***p* value** |
| --- | --- | --- | --- | --- | --- |
| **Any Nodes (228)** | | | | | |
| **Negative (136)** | 10·3  (6·1 – 17·2) | Referent |  | Referent |  |
| **Positive (92)** | 23·3  (15·7 – 33·8) | 2·58  (1·28 – 5·19) | 0·008 | 2·31  (1·14 – 4·68) | 0·020 |
| **Total Nodes** |  | 1·22  (1·11 – 1·34) | <0·001 | 1·20  (1·09 – 1·32) | <0·001 |
| **N stage (228) V7** | | | | | |
| **N0**  **(136, 60^)** | 10·3  (6·1 – 17·2) | Referent |  | Referent |  |
| **N1**  **(44, 19%)** | 17·7  (8·8 – 22·8) | 1·78  (0·71 – 4·46) | 0·219 | 1·64  (0·65 – 4·11) | 0·295 |
| **N2**  **(20, 9%)** | 21·1  (8·4 – 47·1) | 2·33  (0·76 – 7·14) | 0·140 | 1·97  (0·63 – 6·15) | 0·245 |
| **N3**  **(28, 12%)** | 33·5  (19·0 – 54·6) | 4·31  (1·84 – 10·10) | <0·001 | 3·86  (1·64 – 9·09) | 0·002 |
| **N stage V8** | | | | | |
| **N0 (136, 60%)** | 10·3  (6·1 – 17·2) | Referent |  | Referent |  |
| **N1a (87, 38%)** | 22·4  (14·7 – 33·2) | 2·45  (1·20 – 5·01) | 0·014 | 2·20  (1·07 – 4·52) | 0·031 |
| **N1b (1· 0·4%)** | - | 21·00  (2·66 – 165·64) | 0·004 | 24·27  (3·05 – 193·34) | 0·003 |
| **N1c (4, 2%)** | 25·0  (4·0 – 87·2) | 2·91  (0·38 – 22·3 | 0·303 | 2·01  (0·26 – 15·77) | 0·507 |
| **Perirectal Nodes (198)** | | | | | |
| **Negative (136)** | 10·3  (6·1 – 17·2) | Referent |  | Referent |  |
| **Positive (62)** | 24·0  (14·9 – 37·2) | 2·71  (1·27 – 5·76) | 0·010 | 2·40  (1·12 – 5·14) | 0·024 |
| **Perirectal Nodes (198)** | | | | | |
| **Negative (136)** | 10·3  (6·1 – 17·2) | Referent |  | Referent |  |
| **Mesorectal without pre-sacral (38)** | 19·7  (9·9 – 37·1) | 2·14  (0·85 – 5·36) | 0·105 | 2·02  (0·81 – 5·07) | 0·133 |
| **Sacral w/wo PR (24)** | 30·4  (15·7 – 53·6) | 3·69  (1·47 – 9·28) | 0·005 | 3·01  (1·17 – 7·72) | 0·022 |
| **Internal Nodes (154)** | | | | | |
| **Negative (136)** | 10·3  (6·1 – 17·2) | Referent |  | Referent |  |
| **Positive (18)** | 35·0  (17·3 – 62·4) | 4·72  (1·79 – 12·45) | 0·002 | 3·91  (1·47 – 10·40) | 0·006 |
| **Inguinal Nodes (169)** | | | | | |
| **Negative (136)** | 10·3  (6·1 – 17·2) | Referent |  | Referent |  |
| **Positive (33)** | 25·5  (13·6 – 44·6) | 3·00  (1·24 – 7·22) | 0·015 | 2·57  (1·05 – 6·30) | 0·038 |

DMF: Distant Metastatic Failure; HR: hazard ratio; CI: confidence interval. Values in parentheses represent patient numbers;

*Univariable Cox regression; †Multivariable Cox regression adjusted for mrT-size; §Involved field compared with those patients deemed node negative.
